# The Parkinson’s disease risk gene cathepsin B promotes fibrillar alpha-synuclein clearance, lysosomal function and glucocerebrosidase activity in dopaminergic neurons

**DOI:** 10.1186/s13024-024-00779-9

**Published:** 2024-11-25

**Authors:** Jace Jones-Tabah, Kathy He, Nathan Karpilovsky, Konstantin Senkevich, Ghislaine Deyab, Isabella Pietrantonio, Thomas Goiran, Yuting Cousineau, Daria Nikanorova, Taylor Goldsmith, Esther del Cid Pellitero, Carol X.-Q. Chen, Wen Luo, Zhipeng You, Narges Abdian, Jamil Ahmad, Jennifer A. Ruskey, Farnaz Asayesh, Dan Spiegelman, Stanley Fahn, Cheryl Waters, Oury Monchi, Yves Dauvilliers, Nicolas Dupré, Irina Miliukhina, Alla Timofeeva, Anton Emelyanov, Sofya Pchelina, Lior Greenbaum, Sharon Hassin-Baer, Roy N. Alcalay, Austen Milnerwood, Thomas M. Durcan, Ziv Gan-Or, Edward A. Fon

**Affiliations:** 1 Neurodegenerative Diseases Group, Department of Neurology and Neurosurgery, McGill Parkinson Program, Montreal Neurological Institute-Hospital, McGill University, Montreal, Québec Canada; 2 Department of Neurology and Neurosurgery, McGill University, Montréal, Canada; 3 Early Drug Discovery Unit (EDDU), Montreal Neurological Institute-Hospital, McGill University, Montreal, Canada; 4 Research Department, Bioinformatics Institute, Saint-Petersburg, Russia; 5 Department of Human Genetics, McGill University, Montréal, Canada; 6 Department of Neurology, College of Physicians and Surgeons, Columbia University Medical Center, New York, NY USA; 7 Département de Radiologie, Radio-Oncologie Et Médecine Nucléaire, Université de Montréal, Montréal, QC Canada; 8 Centre de Recherche de L’Institut Universitaire de Gériatrie de Montréal, Montréal, QC Canada; 9 Sleep Unit, Department of Neurology, National Reference Center for Narcolepsy, Gui-de-Chauliac Hospital, CHU Montpellier, University of Montpellier, Montpellier, France; 10 Neuroscience Axis, CHU de Québec – Université Laval, , Quebec City, G1V 4G2 Canada; 11 Department of Medicine, Faculty of Medicine, Université Laval, Québec, QC G1V 0A6 Canada; 12 Institute of the Human Brain of RAS, St. Petersburg, Russia; 13 First Pavlov State Medical, University of St. Petersburg, Saint-Petersburg, Russia; 14 Sackler Faculty of Medicine, Tel Aviv University, Tel Aviv, Israel; 15 The Joseph Sagol Neuroscience Center, Sheba Medical Center, Tel Hashomer, Ramat Gan, Israel; 16 Department of Neurology, The Movement Disorders Institute, Sheba Medical Center, Tel Hashomer, Ramat Gan, Israel; 17 Neurological Institute, Tel Aviv Sourasky Medical Center, Tel Aviv, Israel

## Abstract

**Background:**

Variants in the *CTSB* gene encoding the lysosomal hydrolase cathepsin B (catB) are associated with increased risk of Parkinson’s disease (PD). However, neither the specific *CTSB* variants driving these associations nor the functional pathways that link catB to PD pathogenesis have been characterized. CatB activity contributes to lysosomal protein degradation and regulates signaling processes involved in autophagy and lysosome biogenesis. Previous in vitro studies have found that catB can cleave monomeric and fibrillar alpha-synuclein, a key protein involved in the pathogenesis of PD that accumulates in the brains of PD patients. However, truncated synuclein isoforms generated by catB cleavage have an increased propensity to aggregate. Thus, catB activity could potentially contribute to lysosomal degradation and clearance of pathogenic alpha synuclein from the cell, but also has the potential of enhancing synuclein pathology by generating aggregation-prone truncations. Therefore, the mechanisms linking catB to PD pathophysiology remain to be clarified.

**Methods:**

Here, we conducted genetic analyses of the association between common and rare *CTSB* variants and risk of PD. We then used genetic and pharmacological approaches to manipulate catB expression and function in cell lines, induced pluripotent stem cell-derived dopaminergic neurons and midbrain organoids and assessed lysosomal activity and the handling of aggregated synuclein fibrils.

**Results:**

We find that catB inhibition impairs autophagy, reduces glucocerebrosidase (encoded by *GBA1*) activity, and leads to an accumulation of lysosomal content. In cell lines, reduction of *CTSB* gene expression impairs the degradation of pre-formed alpha-synuclein fibrils, whereas *CTSB* gene activation enhances fibril clearance. In midbrain organoids and dopaminergic neurons treated with alpha-synuclein fibrils, catB inhibition potentiates the formation of inclusions which stain positively for phosphorylated alpha-synuclein.

**Conclusions:**

These results indicate that the reduction of catB function negatively impacts lysosomal pathways associated with PD pathogenesis, while conversely catB activation could promote the clearance of pathogenic alpha-synuclein.

**Supplementary Information:**

The online version contains supplementary material available at 10.1186/s13024-024-00779-9.

## Introduction

Parkinson’s disease (PD) is characterized by both the degeneration of dopaminergic neurons in the substantia nigra and by the accumulation of Lewy bodies, proteinaceous inclusions composed largely of misfolded and aggregated α-synuclein (α-syn) [1]. Mutations that increase protein levels of α-syn or its propensity to aggregate contribute substantial genetic risk to PD [2, 3], supporting the predominant hypothesis that α-syn aggregation is a key step in the pathological cascade leading to neurodegeneration in PD. The lysosome serves as the principal site for degradation of aggregated α-syn [4–6], and mutations in lysosomal genes also represent a substantial genetic risk for PD [7]. Thus, there is great interest in understanding the lysosomal pathways that mediate α-syn clearance. Cathepsin B (catB, encoded by the *CTSB* gene) is a proteolytic enzyme of the cysteine cathepsin family with endo- and exo-peptidase activity that is normally localized to the lysosomal lumen [8]. CatB has been implicated both in the lysosomal degradation of α-syn and as a genetic risk factor for PD. In the present study we further elucidate the relationship between *CTSB* variants and PD risk and use cell lines, human dopaminergic neurons and midbrain organoids to demonstrate that catB modulates lysosome function and the clearance of α-syn aggregates.

The importance of lysosomal function in PD is well established by both functional and genetic studies [7]. Recently, genome-wide association studies (GWAS) have identified significant association between variants in the *CTSB* genetic locus and the risk of PD generally [9] and specifically in carriers of pathogenic *GBA1* variants [10]. In addition to genetic evidence linking *CTSB* to PD, catB protein or activity levels are reduced in several cellular models of PD. For example, pathological α-syn species have been shown to impair catB trafficking to the lysosome [11], while iPSC derived neurons harboring mutations in *SNCA* or *GBA1* exhibited reduced catB activity [12, 13]. Additionally, knockout of the PD risk gene *TMEM175* impairs catB activity by destabilizing lysosome pH [14, 15], while mutations in *LRRK2,* the most common cause of familial PD, have been shown to suppress catB expression or activity in the lysosome [16, 17]. Thus, several lines of evidence suggest that disrupted catB function could play a role in PD pathogenesis.

One potential mechanism linking catB to PD is through its ability to cleave both monomeric and aggregated forms of α-syn, which has been demonstrated in vitro [18–20]. However, while this could argue for a protective role of catB against synucleinopathy, the α-syn truncations produced by in vitro catB cleavage exhibit an increased propensity to aggregate [21] and although lysosome function is essential for degradation of fibrillar α-syn [22], it has also been suggested that catB activity contributes to α-syn toxicity in some cellular models [23]. Moreover, catB has been linked to the α-syn dependent activation of inflammatory pathways [24] and is a key regulator of cell death in many cellular contexts [25]. Thus, there are compelling arguments to be made in favor of both protective and potentially pathogenic actions of catB in the etiology of PD and its specific role remains to be elucidated.

Here, we aim to both clarify the genetic evidence pertaining to how *CTSB* variants may contribute to PD etiology, and to functionally characterize the role of catB in relation to lysosome function and α-syn clearance. We first provide genetic evidence that PD-associated *CTSB* variants decrease expression levels of the enzyme. Second, by pharmacologically and genetically modulating catB expression or activity in cell lines and human dopaminergic neurons, we demonstrate that catB is required for lysosomal functions including glucocerebrosidase activity and contributes to clearance of fibrillar α-syn. These findings argue in favor of a protective effect of catB in PD.

## Materials and methods

### Fine mapping and eQTL analysis of CTSB variants

To identify the most likely variant driving the PD-association in the *CTSB* locus, we performed analyses using the summary statistics from the most recent PD GWAS [9] and multiple bioinformatic tools. First, to examine whether there are multiple independent associations in this locus, we used genome-wide complex trait conditional and joint analysis (GCTA-COJO) [26], using default parameters. For downstream analyses, we generated a linkage disequilibrium (LD) matrix for the *CTSB* locus using PLINK 1.9 [27], including all variants within ± 1Mbp from the top variant. Then, we performed fine-mapping of the *CTSB* locus to nominate the most likely driving variants using FINEMAP [28], with minor allele frequency (MAF) threshold > 0.01. Expression quantitative trait locus (eQTL) analysis was performed using colocalization (COLOC) [29], which examines whether the same variants associated with the trait (PD) are also associated with gene expression. QTLs were tested in a total of 109 tissues and cells (Supplementary Table 1) To further explore the link between genetic variants, QTLs and PD we used Summary-data-based Mendelian Randomization (SMR), which uses Mendelian randomization to suggest potential causality, followed by heterogeneity in dependent instruments (HEIDI) to distinguish between pleiotropy (or causality) and LD [30].

### Rare variant analysis

Rare variant analysis was performed on 5,801 PD cases and 20,427 controls across six cohorts (Supplementary Table 2). All patients were diagnosed by movement disorder specialists according to the UK brain bank criteria [31] or MDS diagnostic criteria [32]. From the AMP-PD and UKBB cohorts we only included participants of European ancestry and excluded any first and second-degree relatives from the analysis. Quality control procedures for AMP-PD and UKBB were performed as previously described in detail [33, 34].

In addition, we conducted sequencing on four distinct cohorts at McGill University (McGill cohort, Columbia cohort, Sheba Medical Center cohort and Pavlov and Human Brain Institutes cohort). We performed sequencing of the *CTSB* gene, including exon–intron boundaries (± 50bps) and the 5’ and 3’ untranslated regions (UTRs) using molecular inversion probes (MIPs) as described previously [35]. The full protocol is available at https://github.com/gan-orlab/MIP_protocol. Library sequencing was performed by the Genome Quebec Innovation Centre on the Illumina NovaSeq 6000 SP PE100 platform. We used Genome Analysis Toolkit (GATK, v3.8) for post-alignment quality checking and variant calling [36]. We applied standard quality control procedures [37]. In brief, only variants with minor allele frequency (MAF) of less than 1% and a minimum quality score (GQ) of 30 were included in the analysis. The average coverage for *CTSB* in cohorts sequenced at McGill was > 4000X with 95% nucleotides covered at 30x (Supplementary Table 3).

To analyze rare variants, we applied the optimized sequence Kernel association test (SKAT-O, R package) [38] with further meta-analysis between the cohorts using metaSKAT package [39]. We performed separate analyses for the whole gene, non-synonymous and functional (nonsynonymous, stop and frameshift variants) and variants with Combined Annotation Dependent Depletion (CADD) scores of ≥ 20 [40]. We adjusted for sex, age and ethnicity in all analyses. We also analyzed whether rare *CTSB* variants affected age at onset of PD.

### Generation of CTSB-KO and SNCA-KO iPSC

The previously characterized AIW002-02 iPSC cell line [41] was used to generate *CTSB* and *SNCA* knockout lines. CRISPR gRNAs were designed using Synthego and the sequences of reagents used are depicted in Supplementary Table 4. The SNCA-KO line was created by using two gRNAs to introduce a 122 bp deletion into exon 1 of the gene. The gRNA sequences were cloned into a Cas9/puromycin expression vector PX459 (Addgene, #48,139) and transfected into iPSCs using Lipofectamine ™ stem reagent (ThermoFisher Scientific). Transfected iPSCs were selected in 0.3 μg/mL puromycin for 72 h and surviving colonies were manually picked and expanded for PCR screening to confirm deletion of the target region. Colonies confirmed to be knockout by PCR screening were further validated by sanger sequencing, and loss of protein was confirmed by Western blot in differentiated neurons.

The *CTSB*-KO cell line was created by HDR using a single gRNA and ssDNA repair template to introduce a stop tag in exon 4 of the gene. Cas9 nuclease, gRNA and a ssDNA repair template for HDR were introduced by Lonza Nucleofection. Edited alleles were detected with ddPCR to select edited clones and deletion was verified by PCR screening followed by sanger sequencing, and finally loss of protein was determined by Western blot (Supplementary Fig. 1).

All lines were subject to quality control as previously described [41] and included verification of pluripotency by immunofluorescent staining for pluripotency markers (Nanog, Tra1-60, SSEA4 and OCT3/4), verification of normal karyotype and verification of normal profile on genome stability test (Supplementary Fig. 1).

### iPSC culture and dopaminergic neuron differentiation

All cell culture reagents used, and media compositions are depicted in Supplemental Table 5. Midbrain neuronal precursor cells (NPCs) and dopaminergic (DA) neurons were generated following previously established protocols [42, 43]. Briefly, iPSCs were dissociated with Gentle Cell dissociation reagent and transferred to uncoated flasks in NPC Induction Media to allow for embryoid bodies (EB’s) to form. EBs were cultured for 7 days and then transferred to polyornithine/laminin coated flasks and grown for another 7 days in NPC induction media. To expand NPCs the EBs were then dissociated into small colonies by trituration in Gentle Cell dissociation media and replated as a monolayer on polyornithine/laminin coated flasks. After reaching confluence, NPCs were harvested and frozen in FBS with 10% DMSO and stored in liquid nitrogen.

To differentiate neurons, NPCs were thawed in NPC Maintenance Media with Y-27632 (ROCK inhibitor) and plated on polyornithine/laminin. NPCs were grown for 5–9 days until confluent. For final differentiation into dopaminergic neurons, NPCs were dissociated using Accutase and plated on polyornithine/laminin in Dopaminergic Differentiation Media. After 5 days, media was supplemented with mitomycin C to remove proliferative cells. DA neurons were maintained by exchanging 1/3 of the culture volume for fresh dopaminergic differentiation media every 5–7 days. Neurons from every batch were assessed by immunofluorescence for expression of Map2 and TH (Supplementary Fig. 2A,B), and only batches achieving at least 50% Map2/TH positivity after 4 weeks of differentiation were used for the experiments included in this manuscript. TH-positivity was determined by high-content imaging immunofluorescence, using an empirically determined intensity threshold to define individual cells as TH-positive.

For high-content imaging experiments neurons were plated on 96-well plates at a density of 15,000 cells per well. For protein and RNA isolation experiments neurons were plated on 6-well plates at a density of 750,000 cells per well. For live-imaging experiments, neurons were plated on 4-chamber imaging dishes at a density of 100,000 cells per well.

### Organoid culture, treatment, and imaging

The patient derived iPSCs with SNCA triplication mutation (3xSNCA) and isogenic SNCA knockout (SNCA-KO) were previously described [44] and provided by Dr. Tilo Kunath. These cells were used to generate midbrain organoids following a protocol previously established in our labs [45, 46]. Three months after organoid induction they were treated with either DMSO (vehicle) or 1 μM CA074me and treatment was maintained for 60 days. All organoids (12 per group) were fixed, cryo-sectioned, and prepared for immunofluorescence using antibodies against Map2, TH, α-syn and pSyn-129 as previously described [45]. Verification of expression of TH and absence of α-syn in SNCA-KO in the organoids used in this study is depicted in Supplementary Fig. 3. Images were acquired using the Leica TCS SP8 confocal microscope and image analysis was performed with an in-house developed script for quantification of immunofluorescent signal in organoids (https://github.com/neuroeddu/OrgQ).

### α-synuclein preformed fibril (PFF) generation and characterization

PFFs were generated from recombinant α-syn monomers as previously described [47, 48]. All PFFs underwent quality control assessment by electron microscopy (Supplementary Fig. 2C,D).

### RPE1 CRISPRa and CRISPRi cell line generation

Human retinal pigment epithelial-1 cells (RPE1) were grown in Dulbecco’s Modified Eagle Medium (Wisent) supplemented with 10% fetal bovine serum (Wisent). To generate CRISPRa and CRISPRi parental cell lines, lentivirus was used to stably transduced RPE1 cells with either pLX_311-KRAB-dCas9 [49] (Addgene #96,918, henceforth referred to as CRISPRi) or EF1a-FLAG-dCas9-VPR [50] (Addgene #114,195, henceforth referred to as CRISPRa) and single clones were selected and characterized to generate monoclonal parental lines stably expressing the CRISPRa and CRISPRi machinery. The gRNA sequences targeting our genes of interest were selected from previously published CRISPRa/i libraries [51] (Supplemental Table 6), synthesized by IDT and cloned into pCRISPRi/a-v2 [51] (Addgene #84,832). Lentivirus was used to stably transduce parental CRISPRa and CRISPRi cell lines which then underwent puromycin selection to generate polyclonal cell lines expressing the gRNA of interest. For each target, several gRNAs were tested and the best performing sequences were selected by assessing target modulation by RT-qPCR analysis of gene expression.

### Drug and PFF treatments

CA074me (Selleckchem) and PADK (Bachem) treatments were performed at the indicated final concentrations with DMSO as vehicle. For PFF experiments in Figs. 2 and 3, a single drug treatment was performed simultaneous with PFF administration, after which media was refreshed every 5–7 days. For lysosomal assays in Fig. 3, drug was administered 5 days prior to the assay unless otherwise specified.

For PFF treatments on RPE1 cells, 50,000 cells were plated on 12-well plates. After 24 h, PFF was added and cells were allowed to continue growing for 48 h before cells were washed with PBS and dissociated with trypsin to remove non-internalized PFFs before being lysed in RIPA buffer.

For PFF high-content imaging assays with PFF, neurons were treated with PFF after 2 weeks of differentiation in 96-well plates. After treatment, media was refreshed every 5–7 days normally. At completion of treatment, cells were washed with PBS and fixed with 4% paraformaldehyde.

### High-content imaging—immunofluorescence

Cells were permeabilized for 10 min with 0.3% saponin (lysosome immunostaining) or 0.2% triton X-100 (pS129- α-syn assay and TFEB assay) in PBS and blocked with 1% BSA, 4% goat-serum and 0.02% triton X-100 in PBS. Antibodies used are described in Supplemental Table 7. High content imaging was performed on an Opera Phenix high-content confocal microscope (Perkin Elmer) and image analysis was performed using Columbus (Perkin Elmer). Data processing was then conducted using R studio as previously described [52]. Briefly, nuclei were first identified by the Hoechst channel, and surrounding soma was identified as Map2-positive region. Relevant secondary stains were then quantified within this Map2-defined region. Single-cell data were then processed using a custom script in R studio to filter objects based on nuclear size, nuclear shape and Map2 staining intensity to identify only the neuronal cells for inclusion in subsequent analysis.

For TFEB localization experiments, nuclei were defined as the DAPI-positive region and cell soma were defined as a region positive for either phalloidin (RPE1 cells) or Map2 (neurons) excluding the DAPI-defined nucleus.

### High content imaging—live cell assays

#### PFB-FDGlu GCase activity assay

Cells in 96-well plates were pre-loaded for 30 min with lysotracker deep red (1:20,000, Invitrogen). Media was then exchanged for FluoroBrite imaging media (Thermo) containing 25uM of PFB-FDGlu (Invitrogen) and cells were then imaged on the Opera Phenix every 15 min for 2 h to monitor GCase activity. Using the Columbus software, lysotracker signal was used to identify cells for quantification of GCase substrate fluorescence, which is depicted as the mean fluorescence per cell.

#### DQ-BSA

Cells were pre-loaded with DQ-BSA (Invitrogen) for the indicated duration in standard culture media. Cells were then stained with lysotracker deep red (1:20,000, Invitrogen) for 30 min and media was exchanged for FluoroBrite and imaging was conducted on the Opera Phenix. Using the Columbus software, lysotracker signal was used to identify cells and DQ-BSA fluorescence intensity was measured.

### AAV-mediated CTSB overexpression

The cDNA sequence of CTSB was inserted in frame with the fluorescent protein amCyan and separated with a P2A cleavage site. This construct was cloned into an AAV expression vector under the control of the human synapsin promoter (Addgene plasmid # 50,465). AAV serotype 9 was generated at the CNP Viral Vector Core and the CERVO Brain Research Center. DA neurons were transduced with AAV expressing CTSB or GFP (control) at an MOI of 5000. PFF treatments were initiated 2 weeks after AAV transduction.

### Western blot

Cultured cells were washed with PBS and collected in RIPA lysis buffer with protease inhibitors. Protein concentration was determined using the Pierce™ BCA Protein Assay Kit (Thermo Scientific™) and proteins were prepared at the desired concentration in 6X Laemmli buffer and heated at 95 °C for 5 min. 20 μg of protein were loaded on polyacrylamide gels, run with SDS running buffer and transferred onto nitrocellulose membranes and blocked for 30 min in 5% skim milk made in 1X PBS with 0.1% Tween. For alpha-synuclein blots membranes were fixed using 4% PFA and 0.1% glutaraldehyde for 30 min before blocking. Blocked membranes were incubated with primary antibody (Supplemental Table 7) at 4 °C overnight followed by HRP-conjugated secondary antibodies for 90 min at room temperature. Protein detection was performed by chemiluminescence using Clarity Western ECL Substrate (Biorad) and Western blots were quantified using ImageJ.

### RNA Extraction and qRT-PCR

RNA isolation was performed using the RNeasy Mini Kit (Qiagen) and cDNA was generated from 500 ng of total RNA by RT-PCR using the MMLV Reverse Transcriptase kit (Thermo) with random hexamer primers. Real-time quantitative PCR was performed using SSoAdvanced SYBR Green Master Mix (Biorad) using recommended primer concentrations and 20 ng of cDNA input per reaction. Primers for specified target genes were designed using NCBI PrimerBlast (Supplemental Table 8).

### Live cell confocal imaging and analysis

Neurons were plated on CELLview™ 4-chamber imaging dishes (Greiner) at 100 k cells per well. After 3 weeks of differentiation neurons were treated with alexa633 labelled PFF and/or CA074me. After 72 h neurons were washed and incubated with 50 nM of Lysotracker™ Green DND-26 (Invitrogen) for 30 m at 37 °C in standard culture media. The dye solution was exchanged for FluoroBrite™ DMEM (Gibco) and plates were immediately imaged. Images were acquired on a custom Andor spinning disc confocal microscope at 100X magnification. Single frames were acquired for cell bodies (488 nM Lysotracker, 647 PFF). For neuronal trafficking movies, frames were acquired every 1. 5 s for a total of 61 frames.

For the analysis of somatic lysotracker and PFF colocalization cell bodies were masked manually using FIJI ImageJ. For each image, Lysotracker and PFF signal were binarized using Otsu automatic thresholding, and binarized co-cluster signal was obtained using the “Image Calculation > > AND” function. Somatic slice densities of Lysotracker, PFF, or co-clusters were calculated via “Analyze Particles”. Finally, percentage of PFF in lysosomes was obtained by normalizing co-cluster particle density to Lysotracker density, and percentage of lysosomes containing PFF was similarly obtained by normalizing co-cluster particle density to PFF particle density.

Lysosomal motility was analyzed using the FIJI ImageJ TrackMate plugin [53, 54]. The generated tracks were then filtered by max track speed and then analyzed using Python.

### Electron microscopy

RPE1 cells grown in Lab-Tek chambers (Nunc) were rinsed in 0.1 M Na Cacodylate buffer and fixed with 2.5% glutaraldehyde in 0.1 M Na Cacodylate for 24 h at 4 °C. Cells were then post-fixed with 1% aqueous osmium tetroxide (Mecalab) for 1 h at 4 °C, and stained with 4% uranyl acetate (EMS) in 70% ethanol for 45 min at 4 °C. After dehydrations in ascending alcohols, cells were embedded in Epon resin (Mecalab), and cut at 75 nm thickness in the ultra microtome. Sections were collected in 200 Mesh cooper grids (EMS) and stained with 4% uranyl acetate for 5 min following by Reynold’s lead citrate for 2 min. Sections were visualized using a transmission electron microscope (Tecnai G2 Spirit Twin 120 kV Cryo-TEM) coupled to a camera (Gatan Ultrascan 4000 4 k × 4 k CCD Camera model 895). The identification of cellular elements was based on standard descriptions [55].

### Statistical analysis

Statistical analysis was conducted in GraphPad Prism9 software. For experiments with iPSC-derived neurons biological replicates were defined as experiments conducted at different times from the same batch of banked NPCs, or as experiments conducted in parallel from different batches of NPCs. A minimum of 3 distinct batches of NPCs were used for each experiment. Statistical comparisons were performed using t-tests (only 2 conditions), Bonferroni-corrected t-tests (more than 2 conditions compared). Significance levels are depicted in figure legends.

## Results

### Variants in CTSB likely drive the association with PD and are associated with CTSB expression in multiple brain regions

**Fig. 1:**
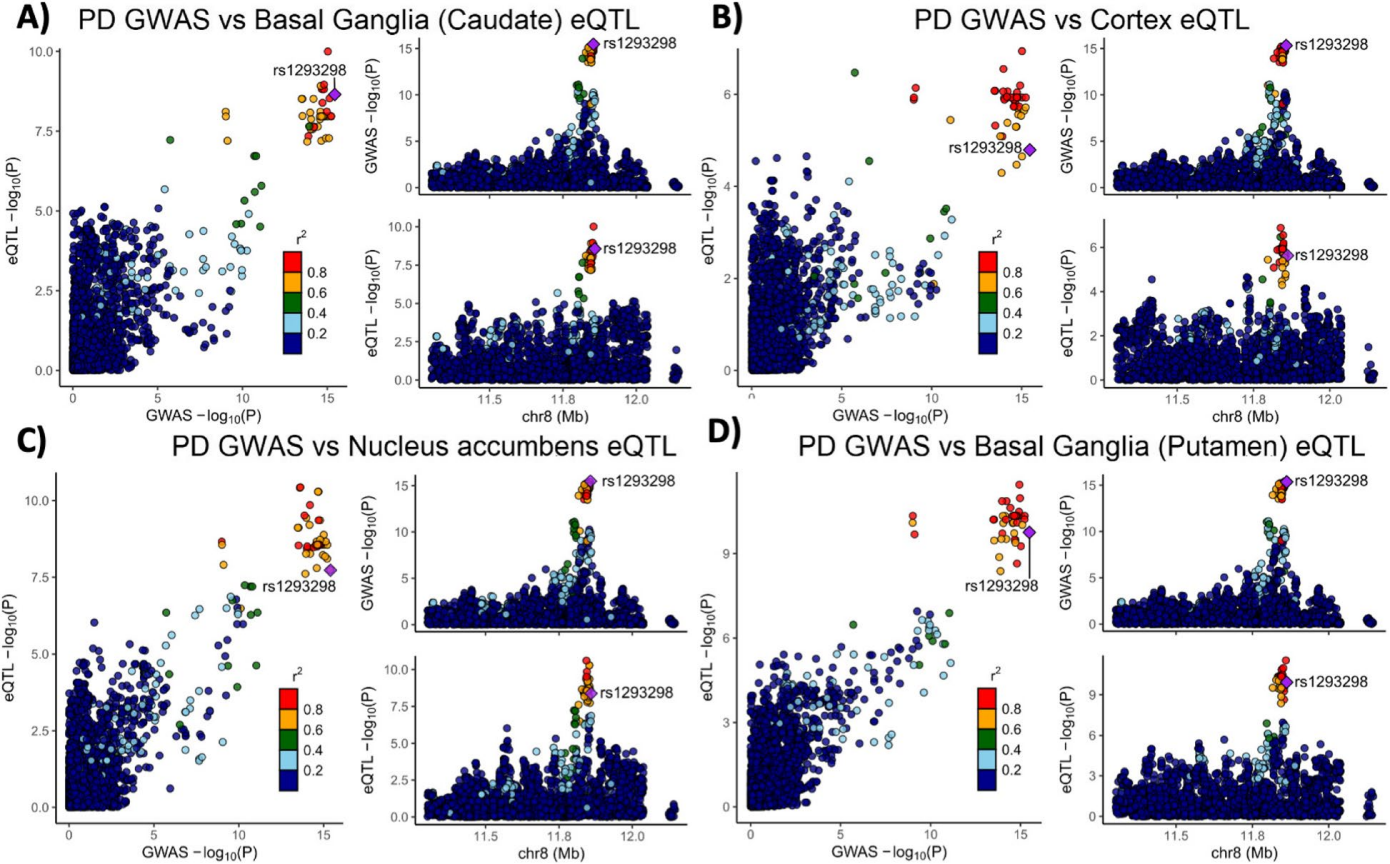
Genetic dissection of the *CTSB* locus in Parkinson’s disease risk. Locus zoom plots depicting the *CTSB* locus (± 500 kb) in Parkinson's disease GWAS with brain eQTLs. The top PD-associated variant (rs1293298) is highlighted in purple, and variants in strong LD (r^2^ > 0.8) are in red. Each panel includes three plots: The left plot in each panel compares the *p* values from the PD GWAS and expression data for each variant. Variants that are in the top right corner of this plot are therefore associated with both risk of PD and *CTSB* expression. The top right plots depict the PD GWAS association in this locus and is identical in all four panels. On the bottom right of each panel, the plot depicts the association between variants in this locus and *CTSB* RNA expression in the relevant tissue. **A** PD GWAS plotted together with Basal ganglia (Caudate) eQTL. **B** PD GWAS plotted together with Cortex eQTL. **C** PD GWAS plotted together with Nucleus accumbens eQTL. **D** PD GWAS plotted together with Basal ganglia (Putamen) eQTL

Variants in the genetic locus containing *CTSB* are significantly associated with risk of PD [9] yet this locus includes multiple other genes including *FDFT1*, *NEIL2*, *GATA4* and it remains uncertain whether *CTSB* itself drives the association. We examined all the variants that are 1 MB upstream or downstream to the top GWAS variant in this locus and using GCTA-COJO, we show that an intronic *CTSB* variant (rs1293298, *p* = 3.41E-16, located in intron 1 of *CTSB* within a potential enhancer region) is the top variant associated with PD risk, without secondary associations. Fine mapping using FINEMAP gave this variant the highest posterior probability (0.127) of being causal, of all nominated variants. Since the top hit variant rs1293298 is located in a non-coding region, its impact is presumed to be on the regulation of gene expression rather than on the protein sequence. Indeed, this variant is in LD with multiple variants in this locus (r^2^ > 0.8) that are associated with *CTSB* expression with H4 posterior probability > 0.8 in multiple brain regions. The associations between genetic variants, PD and *CTSB* expression in PD-relevant brain regions such as basal ganglia, cortex and nucleus accumbens are depicted in Fig. 1. In particular, the minor allele of the rs1293298 *CTSB* variant linked to PD in GWAS exhibits a protective effect against PD and is associated with elevated expression levels of *CTSB* in brain tissues relevant to the disease (Fig. 1A-D). Analysis using SMR and HEIDI suggests that the QTLs in *CTSB* are potentially causally linked to PD with p HEIDI > 0.05 in multiple tissues (i.e., we could not reject the null hypothesis that there is a single causal variant affecting both gene expression and risk for PD). All results from the GCTA-COLOC, FINEMAP, SMR and HEIDI analyses are detailed in Supplementary Tables 9–11.

These common *CTSB* variants occur in non-coding regions and likely exert their effects through altering expression levels. However, given the evidence that protective *CTSB* variants are associated with increased mRNA expression levels, we hypothesized that loss of function coding variants in *CTSB* would be likely to promote PD risk. We conducted rare variant analysis in 5,801 PD cases and 20,427 controls from six cohorts (Supplementary Table 2). We observed a nominal association between all rare variants and variants with high CADD score and PD risk in the Sheba cohort (*p* = 0.03 and *p* = 0.049, respectively). However, upon examining other cohorts and conducting a meta-analysis we did not find any additional associations (Supplemental Table 12). Additionally, we studied the role of rare *CTSB* variants on the age of PD onset. We found nominal association between functional variants and age at onset in the McGill cohort (*p* = 0.044) and in the meta-analysis for functional and non-synonymous variants (*p* = 0.048 and *p* = 0.043, respectively). All these results should be interpreted with caution as no *p*-values survived multiple comparisons.

### CatB inhibition promotes α-syn aggregation in dopaminergic neurons

**Fig. 2:**
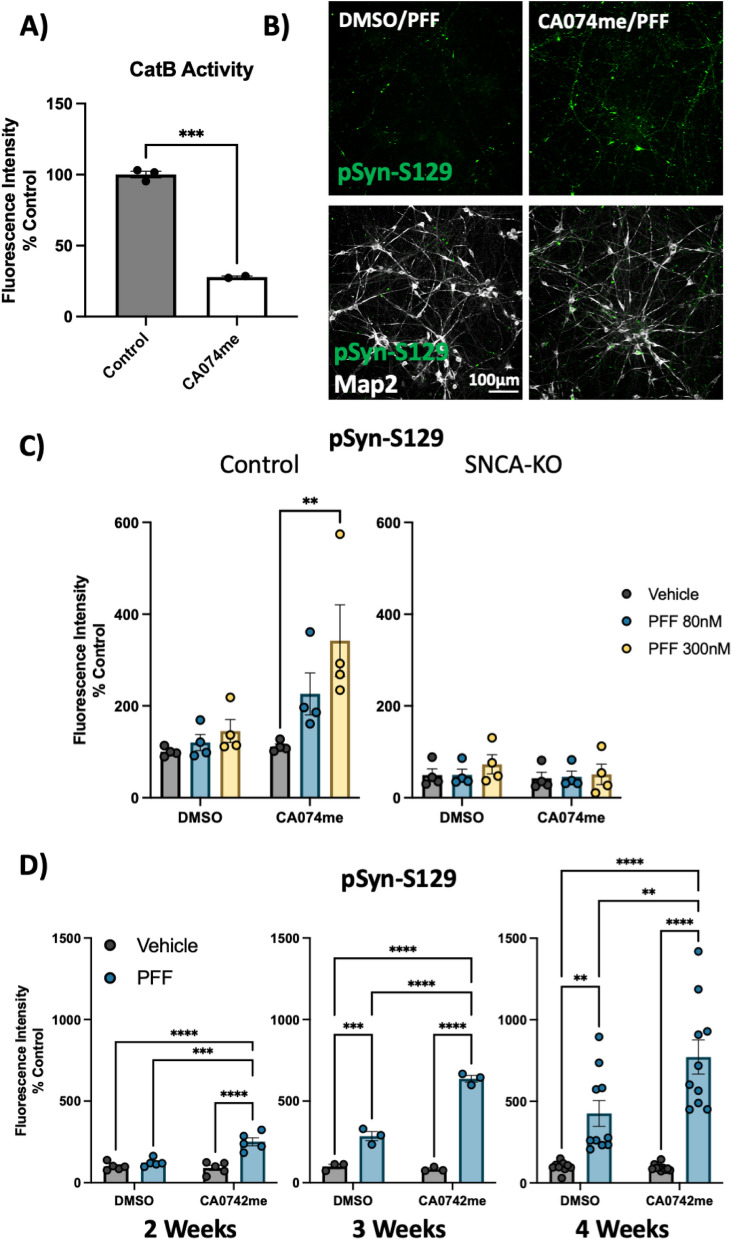
Cathepsin B inhibitors potentiate the effect of α-syn PFFs on dopaminergic neurons: **A**) CatB activity measured by fluorogenic assay in DA neurons treated with 1 μM CA074me. **B** Representative immunofluorescent images from high-content confocal imaging of DA neurons treated with CA074me (1 μM) and/or α-syn PFFs (300 nM) and stained for Map2 and pSyn-S129. **C** Quantification of pSyn-S129 in Map2-positive cells 3-weeks after PFF and/or CA074me treatment in either Control (AIW002-2) DA neurons or isogenic neurons lacking endogenous α-syn (AIW002-2 SNCA-KO). **D** Quantification of pSyn-S129 in control DA neurons 2, 3, or 4 weeks after PFF (300 nM) and/or CA074me treatment. Bonferroni-corrected t-tests, ** *p* < 0.01, *** *p* < 0.001, **** *p* < 0.0001

To functionally interrogate the role of catB in the handling of α-syn fibrils, we generated iPSC-derived DA neurons [41, 42] and treated them with pre-formed α-syn fibrils (PFFs) and the catB inhibitor CA074me [56] at a dose (1 μM), shown to significantly reduce catB activity (Fig. 2A). Exposure to PFFs promotes endogenous α-syn aggregation which can recapitulate many features of Lewy pathology including the accumulation of S129-phosphorylated α-syn (pSyn-S129) [57]. We used immunofluorescence and high-content confocal imaging to quantify pSyn-S129 using Map2 as a counterstain to define the region of neuronal cell bodies and proximal projections (Fig. 2B). At 3-weeks post-treatment PFF dose-dependently induced pSyn-S129 in DA neurons treated with CA074me but not vehicle (DMSO) (Fig. 2C). This pSyn induction was abolished in DA neurons lacking endogenous α-syn (SNCA-KO) (Fig. 2C), indicating that α-syn seeding, rather than phosphorylation of PFFs themselves gave rise to this pSyn-S129 signal. Next, using the 300 nM dose of PFF we tested a range of treatment durations and observed that after 2, 3 or 4 weeks, a single treatment with CA074me administered at the time of PFF exposure increased the abundance of pSyn-S129 (Fig. 2D). CA074me did not affect total α-syn levels (Fig S4A) and no loss of TH + DA neurons was observed 4 weeks post-treatment (Fig S4B). Similar potentiation of pSyn induction was observed with PADK, a distinct catB inhibitor (Fig S4C,D) reinforcing that this likely reflects on-target activity of catB inhibition.

### CatB inhibition induces lysosome dysfunction in dopaminergic neurons

**Fig. 3:**
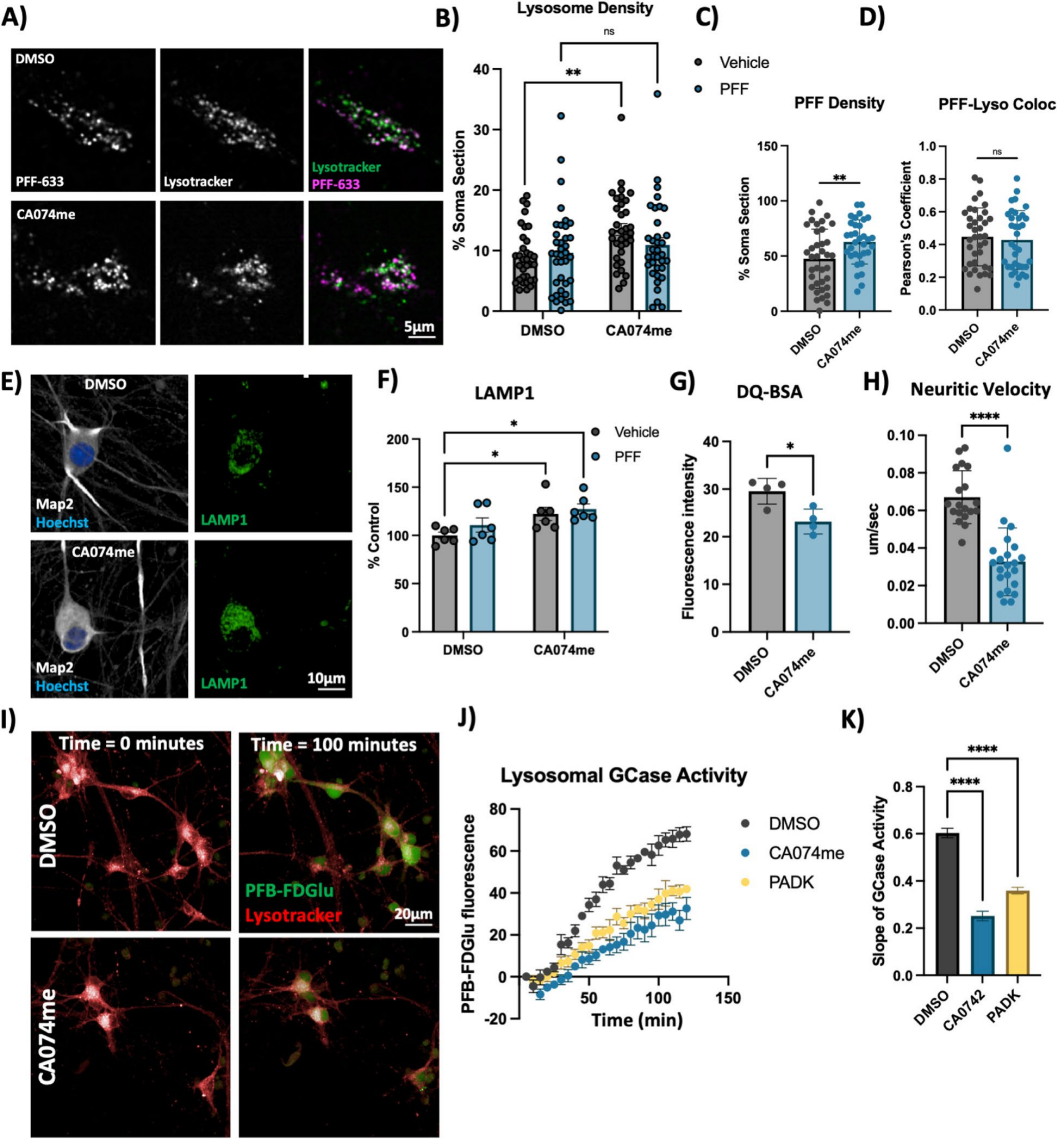
Cathepsin B inhibition increases lysosome abundance but impairs function in dopaminergic neurons. **A** Representative live-cell confocal images of neuronal cell bodies stained with lysotracker-green 72-h after exposure to alexa-633 labelled α-syn PFFs (80 nM). **B** Lysosome density per cell body, measured as the percentage of lysotracker-positive area per cell soma. **C** PFF density per cell body, measured as the percentage of PFF-633-positive area per cell soma. **D** Colocalization of lysotracker and PFF-633 measured using Pearson’s coefficient per cell soma. **E** Representative immunofluorescent images from high-content confocal imaging of DA neurons treated with CA074me (1 μM) and/or α-syn PFFs (300 nM) for 3 weeks and stained for Map2 and LAMP1. **F** Quantification of LAMP1 in Map2-positive cells. **G** Lysosomal degradative capacity measured by fluorescence intensity of DQ-BSA fluorogenic probe 24-h after dye loading. **H** Quantification of lysosome velocity in neurites measured by live-cell confocal imaging and quantified using TrackMate. Points represent individual quantified image fields derived from 6 independent experiments. **I** Representative images of neurons stained with lysotracker deep-red and PFB-FDGlu fluorescent signal at baseline and 100 min after dye-loading. **J** Quantification of PFB-FDGlu fluorescence per cell in DA neurons pre-treated for 24-h with CA074me or PADK. **K** Quantification of the slope of PFB-FDGlu fluorescence versus time. T-test or Bonferroni-corrected t-tests, * *p* < 0.05, ** *p* < 0.01, **** *p* < 0.0001

Extracellular α-syn aggregates are taken into neurons by a variety of mechanisms and are rapidly trafficked to lysosomes [58, 59]. To determine whether CA074me treatment altered the trafficking of PFFs into lysosomes or their persistence there, we performed live cell confocal imaging of DA neurons exposed to alexa-633 tagged PFFs (PFF-633) for 72 h and then stained with lysotracker (Fig. 3A). CA074me increased the overall lysosome content in cells not treated with PFF (Fig. 3B) and also increased the density of PFF-633 fluorescent puncta per cell (Fig. 3C) but colocalization of PFF-633 with lysosomes was unchanged (Fig. 3D). We interpret these observations as indicating that although the abundance of both lysosomes and PFF-633 within each cell is slightly elevated in CA074me treated neurons, the proportion of PFF-633 trafficked to lysosomes is unaffected.

Next, we further characterized how catB inhibition affected lysosome abundance and function. Similar to lysotracker, the abundance of the lysosomal membrane protein LAMP1 was increased after CA074me treatment, independent of concurrent PFF exposure (Fig. 3E, F). However, the degradative capacity of lysosomes (measured using the fluorogenic probe DQ-BSA) was reduced (Fig. 3G). The speed of lysosomal trafficking in neuritic projections was also reduced following CA074me (Fig. 3H). Lastly, given the genetic interaction between variants in *CTSB* and *GBA1* in PD risk [10] and that catB has been found to regulate glucocerebrosidase (GCase) activity in HEK293 cells [60], we assessed the impact of catB inhibition on lysosomal GCase activity in DA neurons using the fluorogenic probe PFB-FDGlu (Fig. 3I-K). CatB inhibition with either CA074me or PADK impaired lysosomal GCase activity (Fig. 3J, K). These observations indicate that despite increasing lysosome abundance, catB inhibition impairs several aspects of lysosome function, including degradative capacity, trafficking and GCase activity.

To determine whether altered lysosome function could be related to the increased abundance of α-syn aggregates, (which has been found to impact lysosomal hydrolase trafficking [11, 13]), we differentiated 3xSNCA and SNCA-KO iPSCs [45] into DA neurons and treated them with CA074me for 3 weeks. We observed that while total levels of α-syn were unchanged by CA074me (Fig S5A,D), LAMP1 was increased in both 3xSNCA and SNCA-KO neurons (Fig S5B,E) indicating the increase in lysosome content is independent of α-syn. Additional staining using the Syn303 antibody, which has been reported to preferentially detect oxidized α-syn species which are often found in pathological aggregates [61], revealed that CA074me treatment increased Syn303 levels (Fig S5C,F) although no S129-pSyn signal above background was detected in these cells. Lastly we observed that CA074me impaired GCase activity in both 3xSNCA and SNCA-KO neurons (Fig S5G,H).

### CTSB repression impairs autophagy and lysosomal function in RPE1 cells

**Fig. 4:**
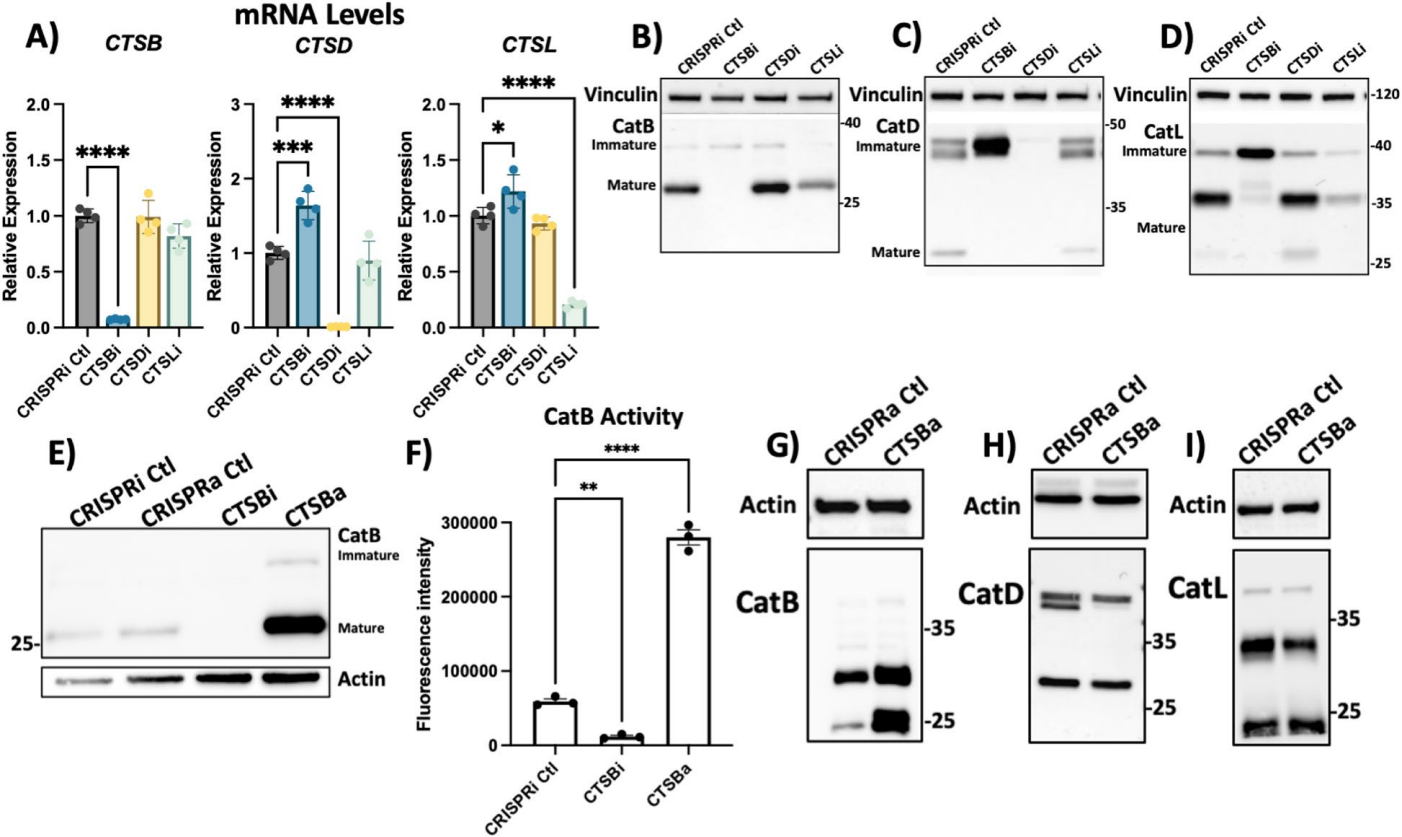
Generation of CTSB, CTSD and CTSL knockdown cell lines. **A** Validation of target knockdown in CRISPRi RPE1 cells. *CTSB*, *CTSD* and *CTSL* mRNA levels were measured by qPCR in control (CRISPRi Ctl), *CTSB*-knockdown (CTSBi), *CTSD*-knockdown (CTSDi) and *CTSL*-knockdown (CTSLi) cell lines. **B** CatB protein levels in control, CTSBi, CTSDi and CTSLi cell lines. **C** CatD protein levels in control, CTSBi, CTSDi and CTSLi cell lines. **D** CatL protein levels in control, CTSBi, CTSDi and CTSLi cell lines. **E** Protein levels of CatB in CRISPRi control, CRISPRa control, *CTSB* knockdown (CTSBi) and *CTSB* upregulation (CTSBa) RPE1 cell lines. **F** CatB activity measured by fluorogenic assay. **G**-**I** CatB, CatD and CatL protein levels in control and CTSBa cells

While CA074me is selective for catB, it has been reported to inhibit other cathepsins, albeit at concentrations greater than those used in this study [62, 63], including *CTSD* and *CTSL* which were previously found to cleave α-syn in vitro [18–20]. To determine how individual cathepsin species regulate lysosome function and contribute to fibrillar α-syn clearance we used CRISPR-interference (CRISPRi) to generate RPE1 cell lines in which *CTSB*, *CTSD*, *CTSL* or α-syn (*SNCA*) were stably repressed (denoted CTSBi, CTSDi, CTSLi and SYNi respectively) as well as CRISPR-activation (CRISPRa) to upregulate *CTSB* (CTSBa). Knockdown or upregulation of target genes was confirmed at transcript and protein levels (Fig. 4A-I). Unexpectedly, we observed that knockdown of CTSB resulted in increased mRNA levels (Fig. 4A), but impaired protein maturation of both catD and catL, indicated by increases in the higher molecular weight immature forms of the protein, and reduction in the proteolytically processed mature forms (Fig. 4C, D). Increased *CTSB* expression by CRISPRa was confirmed to lead to increased mature catB protein (Fig. 4E) and increased catB activity (Fig. 4F). *CTSB* activation also appeared to increase the protein levels of catD but not catL (Fig. 4G-I).

**Fig. 5:**
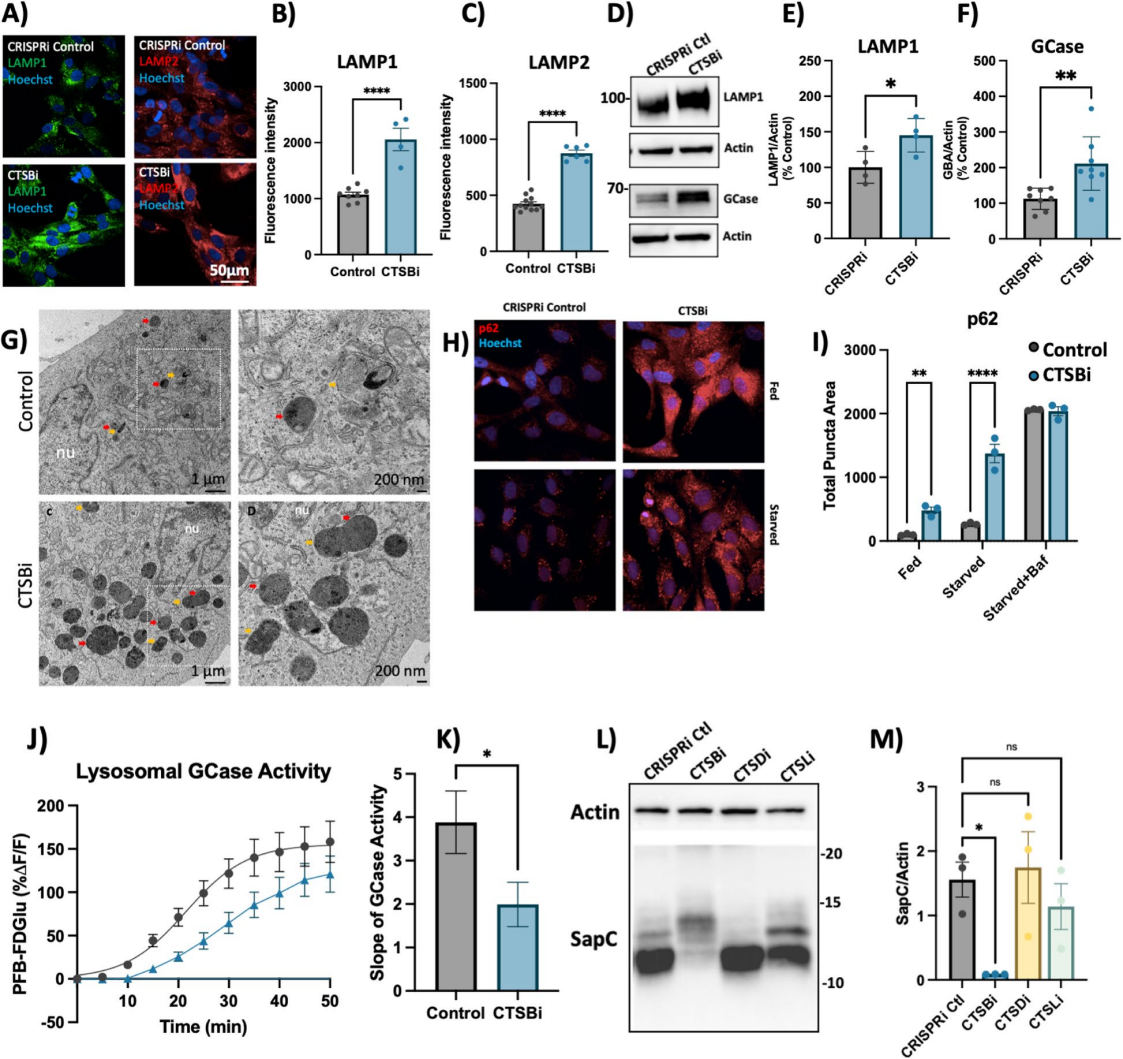
CTSB repression increases lysosome abundance but impairs function in RPE1 cells. **A**-**C** Representative images and quantifications of LAMP1 and LAMP2 immunofluorescence in CRISPRi control and CTSBi RPE1 cells. **D**-**F** Representative western blots and quantifications depicting protein levels of LAMP1 and GCase/*GBA* relative to actin. **G** Electron microscopy images of CRISPRi and CTSBi RPE1 cells. Electron-dense multivesicular bodies-like structures are indicated by red asterisk, lysosomes by orange asterisk, secondary lysosomes by green asterisk, whereas mitochondria are indicated by blue asterisk. **H**, **I** Representative immunofluorescent images and quantification of p62 area per cell in RPE1 cell lines (CRISPRi control – grey bars, and CTSBi – blue bars) under fed, 16-h starvation and 16-h starvation with bafilomycin conditions. **J** GCase activity measured as PFB-FDGlu fluorescence intensity over time. **K** Quantification of the slope of the PFB-FDGlu fluorescence versus time curves. **L**, **M** Representative saposin C (sapC) western blot and quantification relative to actin loading control. Bonferroni-corrected t-tests, * *p* < 0.05, *** *p* < 0.001. T-test or Bonferroni-corrected t-tests, * *p* < 0.05, ** *p* < 0.01, **** *p* < 0.0001

Similar to what we observed in CA074me treated DA neurons, we found that CTSBi cells had significantly increased abundance of LAMP1 and LAMP2 immunofluorescent puncta (Fig. 5A-C), increased LAMP1 and GCase protein levels (Fig. 5D-F) and increased number and size of electron dense lysosome-like structures (including lysosomes and multivesicular bodies) observed by electron microscopy (Fig. 5G). To determine whether impairment in autophagic flux could contribute to the accumulation of lysosomes, we measured the abundance of p62 puncta under fed and starved conditions, and in the presence of bafilomycin (to inhibit lysosomal clearance of autophagosomes). CTSBi resulted in increased abundance of p62 puncta under fed and serum-starved conditions, but not in the presence of bafilomycin (Fig. 5H, I) suggesting an impairment in the clearance of autophagosomes. Similarly, we observed accumulation of the autophagy-associated proteins LC3B and p62 by western blot in CTSBi cells (but not CTSDi or CTSLi), in the absence of serum starvation (Fig S6A,B).

Despite this increase in lysosome content and increased GCase protein levels (Fig. 5D, F), the activity of lysosomal GCase per cell was significantly reduced by *CTSB* knockdown (Fig. 5J, K). One potential mechanism linking CatB to GCase activity is via the cleavage of pro-saposin into saposin C (SapC), which acts as a co-activator of GCase [60]. Using an antibody that recognizes the SapC fragment of the pro-saposin peptide (PSAP) we found that in CTSBi, but not CTSDi or CTSLi cells, the levels of SapC were significantly reduced (Fig. 5L, M), while uncleaved pro-saposin species were increased (Fig S6C). This supports a prior finding that CatB is required for PSAP processing into SapC [60] and may contribute to the reduction in GCase activity.

In addition to impaired clearance of autophagosomes, a second factor that could contribute to increased lysosome abundance is increased lysosome biogenesis. In fact catB has previously been reported to regulate activation of the transcription factor TFEB [64, 65]. Given that mRNA levels of *CTSD* and *CTSL* were already noted to be increased in CTSBi cells (Fig. 4A), we suspected a potential role for increased TFEB activity. Indeed, we found that in CTSBi cells, nuclear localization of TFEB was increased in non-starved cells (Fig S6D, E) and mRNA levels of several lysosomal genes (including *LAMP1*, *GBA* and *MCOLN1*) were transcriptionally upregulated (Fig S6F). These results taken together indicate that a combination of impaired lysosomal enzyme activity, impaired lysosome turnover and upregulated lysosome biogenesis contribute to the increased abundance of dysfunctional lysosomes in RPE1 cells lacking *CTSB*.

### CTSB levels regulate PFF clearance in RPE1 cells

**Fig. 6:**
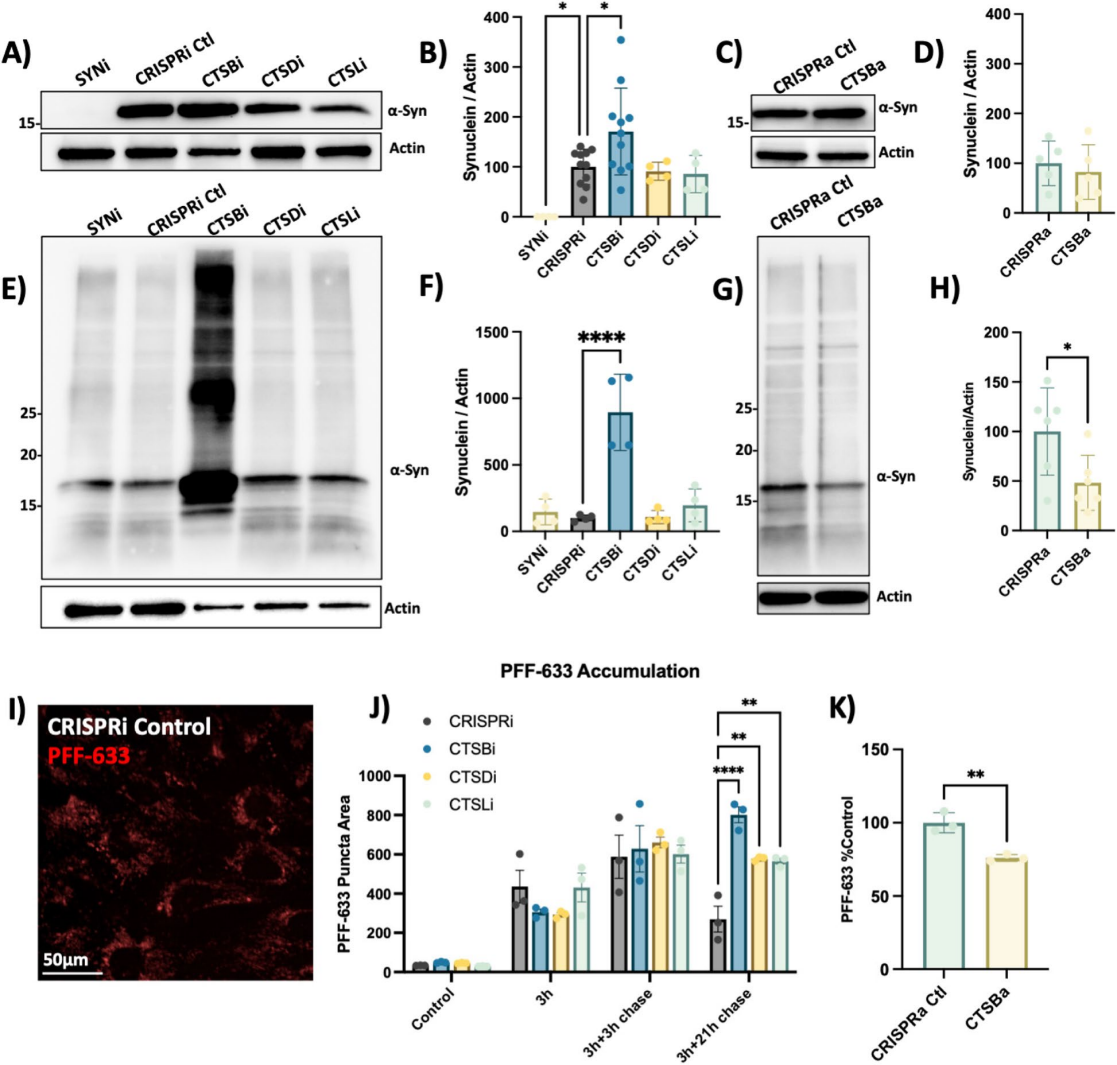
*CTSB* but not *CTSD* or *CTSL* repression impairs PFF clearance in RPE1 cells. **A** Representative western blot depicting protein levels of α-syn and actin. **B** Western blot quantification depicting protein levels of α-syn relative to actin expressed as percentage of CRISPRi control. **C** Representative western blot depicting protein levels of α-syn and actin. **D** Western blot quantification depicting protein levels of α-syn relative to actin expressed as percentage of CRISPRa control. **E** Representative western blot depicting protein levels of α-syn and actin 48-h after treatment of RPE1 cell lines with 300 nM of α-syn PFFs. **F** Western blot quantifications depicting levels of α-syn (quantification of whole lane) relative to actin in PFF treated RPE1 cells. **G** Representative western blot depicting protein levels of α-syn and actin 48-h after treatment of RPE1 cell lines with 300 nM of α-syn PFFs. **H** Western blot quantifications depicting levels of α-syn (quantification of whole lane) relative to actin in PFF treated RPE1 cells. **I** Representative image of CRISPRi control RPE1 cells 48-h after treatment with alexa-633 tagged α-syn PFFs (80 nM). **J**, **K** Quantification of PFF-633 fluorescent intensity per cell in RPE1 cell lines. T-test or Bonferroni-corrected t-tests, * *p* < 0.05, ** *p* < 0.01, *** *p* < 0.001, **** *p* < 0.0001

We next examined the effect of cathepsin knockdown on α-syn protein levels. Endogenous α-syn protein was undetectable in SNCAi cells and elevated in CTSBi cells (Fig. 6A, B). In contrast, CTSBa had no effect on endogenous α-syn protein (Fig. 6C, D). Strikingly, when these cells were exposed to exogenous PFFs for 48 h, CTSBi cells (but not CTSDi or CTSLi) exhibited significantly increased abundance of α-syn, including high molecular weight species, compared to control cells (Fig. 6E, F). Conversely, CTSBa had the opposite effect, modestly reducing the levels of aggregated α-syn (Fig. 6G, H). Moreover, increased α-syn levels were also observed in control or SNCAi cells treated with CA074me (Fig S7) indicating that this increase reflects either increased cellular uptake or failed clearance of the PFFs, rather than new aggregate seeding.

To determine whether the uptake or clearance of PFFs was affected, we used Alexa-633 fluorescently labelled PFFs and conducted a pulse-chase experiment to monitor the uptake and subsequent clearance of PFFs (Fig. 6I, J). During a 3 h exposure, or 3-h exposure with short washout (3 h chase), the PFF-633 levels per cell were similar across cell lines. However, when washout was extended to 21-h CTSBi cells retained more PFF-633 (Fig. 6J), suggesting impaired clearance. CTSDi and CTSLi also appeared to impair PFF clearance in this assay but to a lesser extent than CTSBi. Similar to what we observed by Western blot, when we exposed CTSBa cells to PFF-633 for 48 h, we observed a reduced accumulation of the tagged PFF (Fig. 6K). Taken together, these findings indicate that CatB regulates the clearance of internalized α-syn aggregates, though this may represent either a direct role for CatB in α-syn degradation, or an indirect effect secondary to broader lysosome impairment under conditions of *CTSB* depletion.

### Knockout of CTSB in human dopaminergic neurons leads to lysosomal dysfunction

**Fig. 7:**
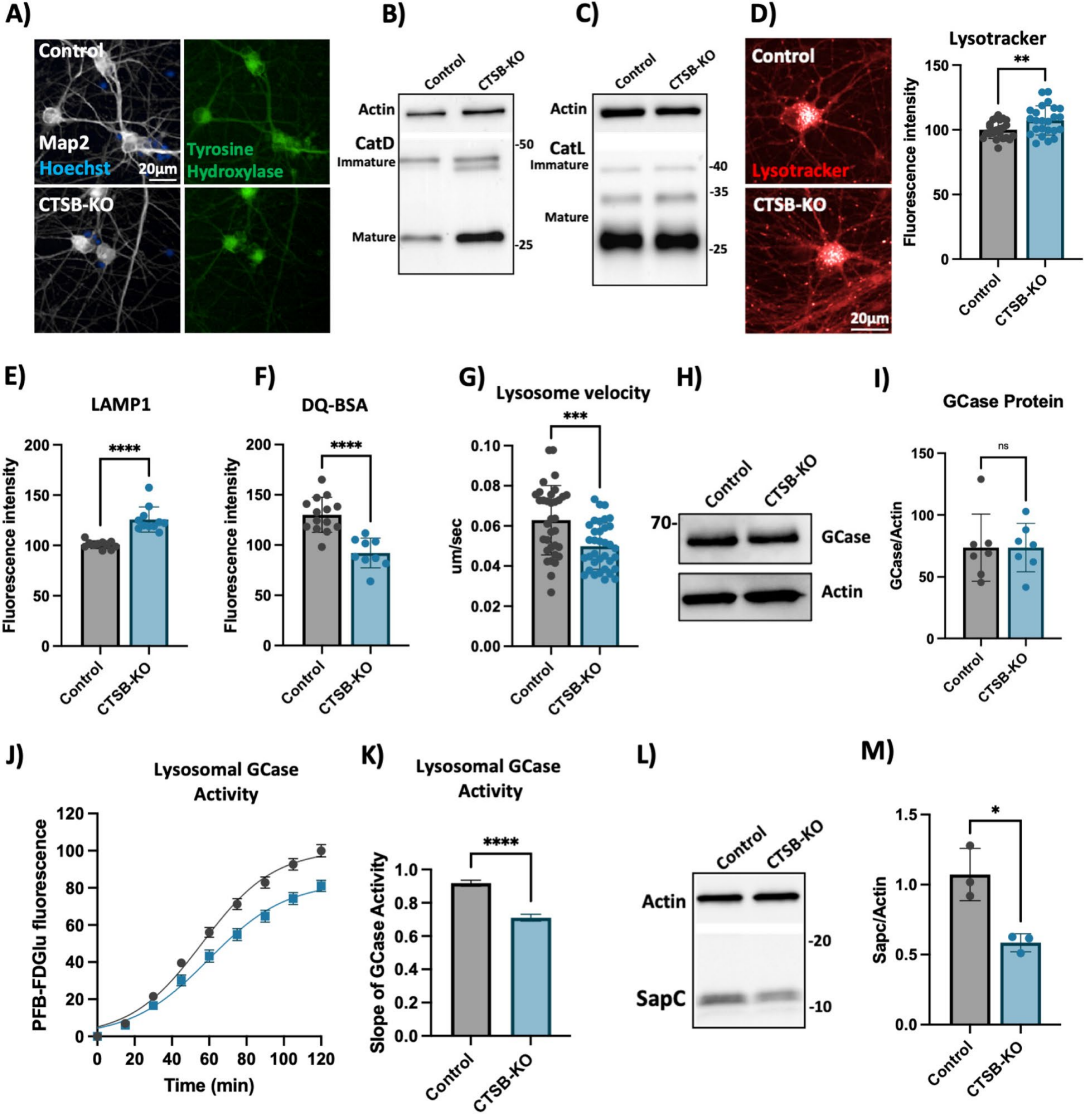
CTSB knockout impairs lysosome function in dopaminergic neurons. **A** Representative immunofluorescent images from high-content confocal imaging of iPSC-derived DA neurons differentiated from control or CTSB-KO iPSCs and stained for Map2, tyrosine hydroxylase (TH) and α-syn. **B** Representative western blot depicting CatD protein levels in control and CTSB-KO neurons. **C** Representative western blot depicting CatL protein levels in control and CTSB-KO neurons. **D** High-content imaging representative image and quantification of lysotracker fluorescence in DA neurons. **E** LAMP1 immunofluorescence per cell in Map2-positive DA neurons. **F** Lysosomal degradative capacity measured by fluorescence intensity of DQ-BSA fluorogenic probe 24-h after dye loading. **G** Quantification of lysosome velocity in neurites measured by live-cell confocal imaging and quantified using TrackMate. Points represent individual quantified image fields derived from 6 independent experiments. **H**, **I** Representative western blot and quantification of GCase and actin in DA neurons. **J** Quantification of PFB-FDGlu fluorescence per cell in DA neurons **K**) Quantification of the slope of PFB-FDGlu fluorescence versus time. **L**, **M** Representative saposin C (sapC) western blot and quantification relative to actin loading control. T-tests, ** *p* < 0.01, *** *p* < 0.001, **** *p* < 0.0001

To further confirm the lysosomal phenotypes that we previously observed in neurons treated with CA074me, we generated *CTSB* knockout iPSCs (CTSB-KO) and differentiated them into DA neurons (Fig. 7A). Both control and CTSB-KO neurons exhibited similar expression patterns of dopaminergic genes (Fig S8A-D), and produced similar percentages of TH-positive neurons within each differentiation (Fig S8E), indicating that loss of *CTSB* did not impact the ability of iPSCs to differentiate into DA neurons. In contrast to CTSBi RPE1 cells, CTSB-KO DA neurons did not exhibit an impairment of CatD or CatL protein processing (Fig. 7B, C). However, similar to CA074me treatment, in CTSB-KO neurons lysosome abundance measured either by lysotracker (Fig. 7D) or LAMP1 immunofluorescence (Fig. 7E) were increased, while degradative capacity (Fig. 7F) and neuritic trafficking velocity (Fig. 7G) were reduced. GCase protein levels were unaffected (Fig. 7H, I) however lysosomal GCase activity was reduced by 20% in CTSB-KO neurons (Fig. 7J, K). We also found that similar to CTSBi RPE1 cells, levels of SapC were significantly reduced in CTSB-KO neurons, which could contribute to the reduction in GCase activity (Fig. 7L, M). Lastly, unlike CTSB-knockdown RPE1 cells, CTSB-KO neurons did not exhibit detectable activation of TFEB (Fig S8F) or transcriptional upregulation of lysosomal genes (Fig S8G) arguing that CatB may regulate TFEB activity and lysosome biogenesis in a cell-type or context-dependent manner.

### CTSB deficiency promotes synuclein pathology in human dopaminergic neurons and midbrain organoids

**Fig. 8:**
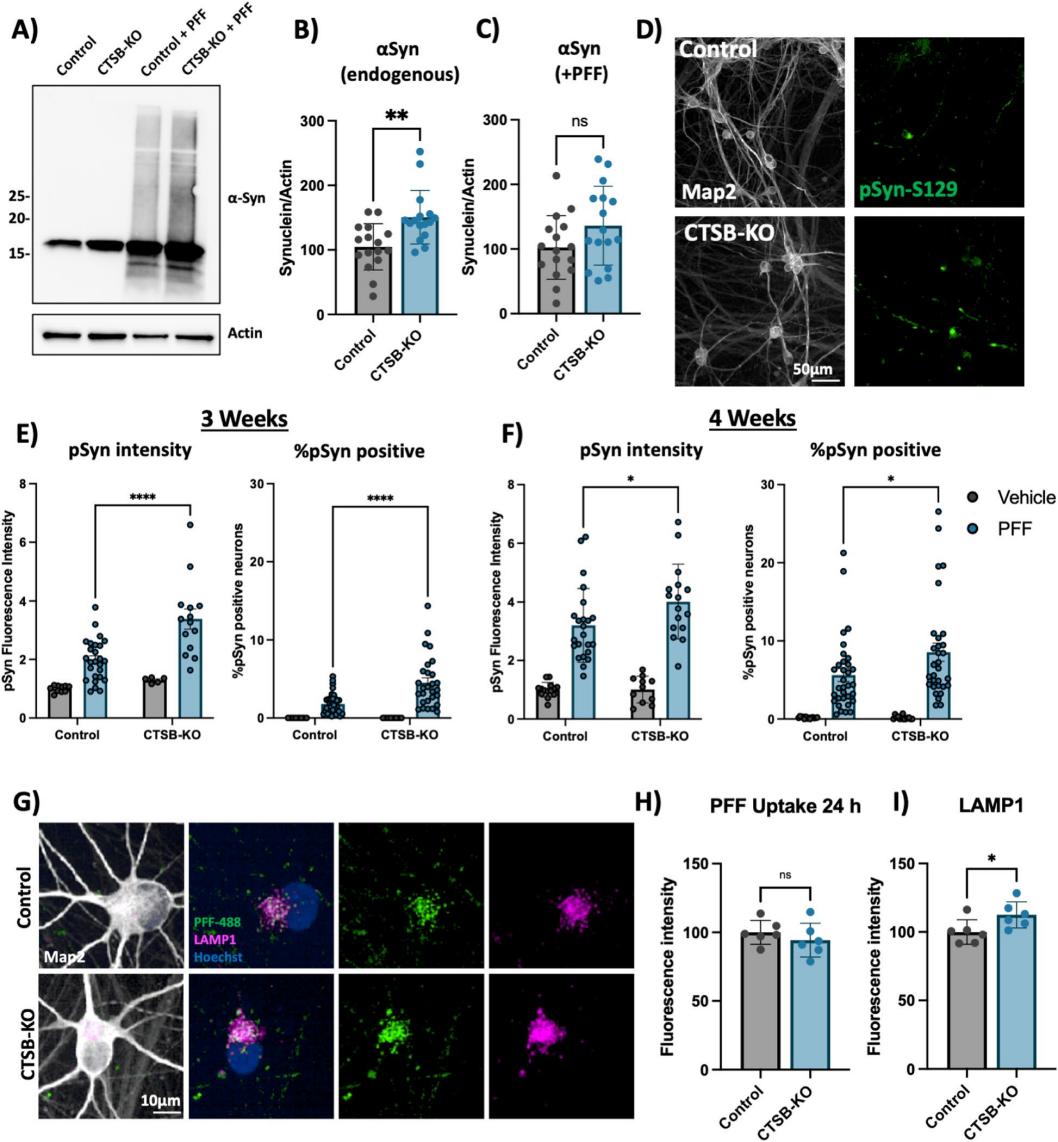
CTSB knockout enhances the effect of α-syn PFFs on dopaminergic neurons. **A** Representative western blot showing levels of α-syn in untreated control or CTSB-KO DA neurons as well as PFF treated DA neurons. **B**, **C** Western blot quantifications depicting protein levels of α-syn relative to actin for endogenous α-syn (**B**) or PFF (**C**), normalized to the respective control. **D** Representative immunofluorescent images from high-content confocal imaging of Control or CTSB-KO DA neurons treated with α-syn PFFs (300 nM) and stained for Map2 and pSyn-S129. E, F) Quantification of pSyn-S129 in Map2-positive cells 3-weeks (**E**) and 4-weeks (**F**) after PFF treatment. Left graphs depict pSyn-S129 fluorescence intensity within Map2-positive cells, and right graphs depict the percentage of Map2-positive cell bodies positive for pSyn-S129 aggregates. **G** Representative immunofluorescent images from high-content confocal imaging of DA neurons treated with alexa-488 tagged PFFs (PFF-488, 80 nM) for 24 h and stained for LAMP1 and Map2. **H** Quantification of PFF-488 fluorescence per Map2-positive cell. **I** Quantification of LAMP1 fluorescence per Map2-positive cell. T-tests or Bonferroni-corrected t-tests, ** *p* < 0.01, **** *p* < 0.0001

Compared to parental control, CTSB-KO DA neurons were found to have modestly elevated levels of endogenous α-syn (Fig. 8A,B). When treated with PFFs for 72 h CTSB-KO neurons did not exhibit higher levels of total α-syn (Fig. 8A, C). However, 3 and 4 weeks after PFF treatment CTSB-KO neurons accumulated significantly more pSyn-S129, and this was evident when measuring either the average pSyn-S129 intensity within Map2-positive neurons, or the percentage of pSyn-positive cell bodies (Fig. 8D-F). The efficiency of PFF uptake (measured by alexa-488 tagged PFF internalization) was unaffected in CTSB-KO neurons (Fig. 8G,H) but as expected LAMP1 was elevated (Fig. 8G,I). Using live-cell confocal microscopy, we observed a modest increase in total PFF-633 levels in CTSB-KO neurons 72 h after treatment (Fig S9A, B), however there was no difference in the trafficking of alexa-633 tagged PFFs to lysosomes as measured by PFF-633 and lysotracker colocalization (Fig S9C). To explore whether CTSB overexpression, or re-expression in the CTSB-KO background could reduce PFF-induced pSyn-S129, we transduced DA neurons with an adeno-associated viral vector (AAV) expressing CTSB along with the fluorescent protein amCyan (Fig S9D). We observed a significant reduction in pSyn-S129 puncta in the CTSB-KO neurons transduced with AAV-CTSB compared to AAV-GFP control (Fig S9E), despite modest levels of CTSB expression achieved with the AAV transgene (Fig S9F), arguing that even small amounts of CTSB expression can promote pSyn-S129 clearance.

**Fig. 9:**
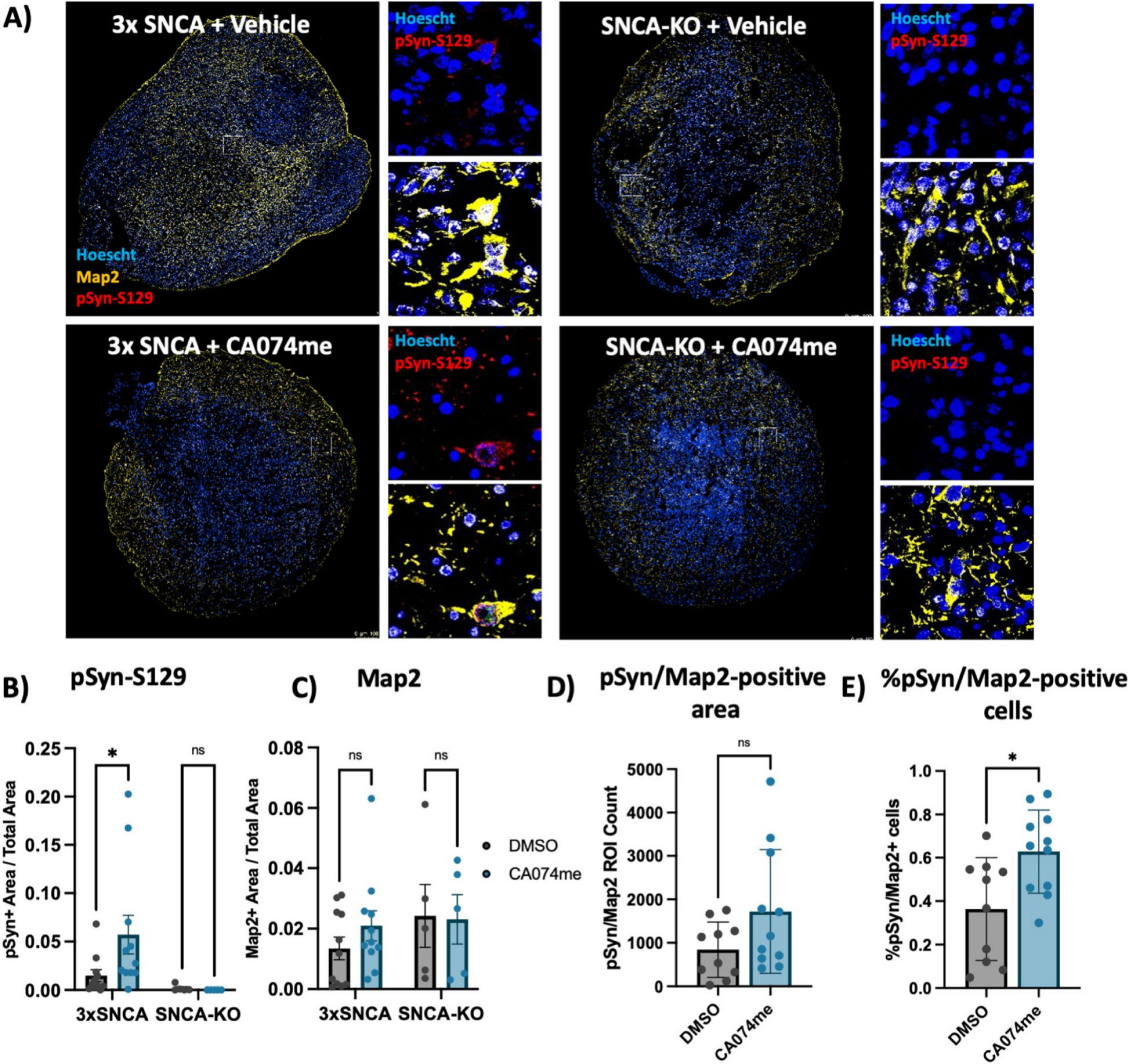
CTSB inhibition promotes pSyn-S129 accumulation in patient-derived midbrain organoids. **A** Representative immunofluorescent images of Map2 and pSyn-S129 in 5-month old SNCA-triplication (3xSNCA) and isogenic SNCA-KO midbrain organoids treated with vehicle (DMSO) or 1 μM CA074me for 60 days. Large images depict representative whole-organoids and high-magnification images depict individual Map2-positive cells. **B** Quantification of the pSyn-S129 positive area of the organoid relative to the total organoid size. **C** Quantification of the Map2-positive area relative to organoid size. **D** Quantification of pSyn-S129- and Map2 double-positive area in 3 × SNCA organoids. **E** Quantification of the percentage of cells positive for both Map2 and pSyn-S129 in 3 × SNCA organoids. Bonferroni-corrected t-tests, * *p* < 0.05

To determine whether the loss of catB function could promote α-syn aggregation independent of PFF exposure, we generated midbrain organoids from patient-derived iPSCs harboring an SNCA triplication mutation (3xSNCA) and an isogenic SNCA-KO line (Fig S3). We have previously described the characterization of these 3xSNCA organoids that spontaneously develop pSyn-S129-positive α-syn aggregates after sustained culture [45]. To determine whether endogenous α-syn aggregation would be affected by catB impairment we treated 3xSNCA or SNCA-KO organoids for 60 days with DMSO (vehicle) or 1 μM CA074me. We observed an increase in the abundance of pSyn-S129 (measured as the area positive for pSyn-S129 relative to total organoid area) (Fig. 9A,B), while treatment had no effect on total neuron content (Map2-positive area) (Fig. 9C). We also observed a trend towards an increase in the total Map2/ pSyn-S129 colocalized area (Fig. 9D), and an increase in the percentage of Map2-positive cells that were positive for pSyn-S129 (Fig. 9E). Co-staining of pSyn-S129 with TH revealed that pSyn signal originated partially, but not exclusively from dopaminergic neurons in both vehicle and CA074me treated organoids (Fig S10). This is similar to what is observed in postmortem PD brain, where pathological inclusions are not limited to dopaminergic neurons [66], and further indicate that loss of catB function increases the burden of pathological α-syn aggregates.

## Discussion

Cathepsin B has previously been suggested to contribute to the degradation of α-syn and genetic variants in the *CTSB* locus are significantly associated with PD, suggesting that this lysosomal protease may play an important role in the disease. Here we provide genetic and functional evidence supporting the crucial involvement of *CTSB* in PD, specifically relating to the function of lysosomes and degradation of α-syn aggregates in dopaminergic neurons.

Firstly, our genetic analysis provides compelling evidence for a causal relationship between common non-coding variants in the *CTSB* gene and both brain expression levels of *CTSB* and PD risk. This genetic analysis indicates that of the variants and genes in the *CTSB* GWAS locus, the association is most likely driven by *CTSB* variants that affect its expression in different brain regions. In particular, the minor allele of the rs1293298 *CTSB* variant, linked to PD in GWAS, exhibits a protective effect against PD and is associated with elevated expression levels of *CTSB* in several brain tissues. This finding is also supported by recent work in which we have used machine learning to nominate the most likely causative genes in each known PD locus, in which *CTSB* was also found to be the top nominated gene [67]. Using the gnomAD database, we also extracted information on loss-of-function (LOF) variants and putative deleterious structural variants. Overall, LOF and structural variants in *CTSB* are rare and we identified only a singular LOF variant in our cohorts. Our burden analysis did not reveal any significant impact of rare *CTSB* variants on PD risk in the meta-analysis, although there was some potential impact on age at onset (Supplemental Table 13, 14). However, the presence of a singular LOF variant did not allow us to assess this impact conclusively.

Interestingly, several cathepsins, including *CTSB*, *CTSD* and *CTSL* have been implicated in the pathogenesis not only of PD, but also additional neurodegenerative disorders including Alzheimers Disease (AD) [68]. Specifically, in AD GWAS, the *CTSB* variant rs1065712 is associated with an increased risk of the disease [69]. The top variant associated with PD (rs1293298), is in LD with the top AD variant, rs1065712 (D' = 1.0, R^2^ = 0.016). This LD suggests a shared genetic architecture between PD and AD at the *CTSB* locus, potentially influencing lysosomal function. However, the direction of the effect differs between the diseases; *CTSB* variants are protective for PD but causative for AD, indicating potentially distinct pathogenic mechanisms for these diseases at this locus.

While several cathepsins appear capable of cleaving α-syn in vitro*, CTSB* alone stand out as a genetic risk factor for PD. Our findings here argue that the mechanism by which *CTSB* variants influence PD risk could be mediated either directly through the ability of catB to cleave and degrade α-syn, which has been previously reported [18–20] or due to the critical role of *CTSB* in maintaining lysosome function more broadly. For example, by using genetic tools to modulate the expression levels of *CTSB*, *CTSD* and *CTSL* in RPE1 cells, we show that in a cellular context, *CTSB* is particularly critical for both the maintenance of lysosome function and clearance of fibrillar α-syn. However, due to secondary effects of CTSB-knockdown on the expression and function of other lysosomal enzymes, we cannot conclude the lack of catB cleavage itself directly leads to increased α-syn accumulation. However, it was recently reported that while many cathepsins exhibit redundancy, the sites within α-syn cleaved by catB are relatively unique, and unlikely to be compensated for by other cathepsins [20]. This lack of redundancy could explain why *CTSB* stands out as a genetic risk factor and an essential mediator of α-syn clearance.

In addition to a potential direct role of catB in degrading α-syn aggregates, we have also observed both genetic and pharmacological catB impairment leads to lysosome accumulation and broad impairment of lysosome functions, including impaired GCase activity. Variants in *CTSB* and *GBA* interact to mediate genetic risk for PD [10] and given the established importance of GCase in mediating risk of synucleinopathy (reviewed in [70]) this raises the question of whether the impaired α-syn clearance observed following catB impairment is partially mediated by secondary GCase impairment. This loss of GCase activity occurs despite an increase in overall lysosome content, and in the case of RPE1 cells, an increase in GCase protein levels. One potential mechanism linking catB to GCase activity is via the ability of catB to cleave pro-saposin into saposin C which acts as a co-activator of GCase [60] and our findings confirm that in dopaminergic neurons loss of catB leads to reductions on SapC, which could contribute to GCase impairment. Future studies will be required to determine the importance of GCase as a mediator of catB -dependent α-syn clearance, as well as the mechanism of interaction between these lysosomal proteins. Interestingly, we also find that the downstream consequence of *CTSB* impairment on lysosome function are cell—type specific. Most notably, we observed that in RPE1 cells but not neurons, CTSB knockdown resulted in impaired maturation of other cathepsins, and activation of TFEB-dependent transcription.

In summary, in the present work we have demonstrated using several cellular models that loss of *CTSB* impairs GCase activity and promotes increased abundance and pathological aggregation of α-syn after exposure to preformed α-syn fibrils. These findings complement genetic evidence that *CTSB* variants associated with increased expression levels are protective against the disease and provides potential mechanistic support for the genetic interaction between *CTSB* and *GBA*. Beyond the direct genetic association, impaired catB expression or activity have also been reported in cellular or animal models associated with PD-risk factors like α-syn/*SNCA*, *GBA*, *TMEM175* and *LRRK2* [11–17, 71]. Together this evidence highlights *CTSB* as an important player in the etiology of synucleinopathies such as Parkinson’s disease, and further study of its biology may help to uncover novel therapeutic approaches to this disease.

## Supplementary Information

Supplementary Material 1. Supplementary Table 1: QTL datasets used for analyses. Supplementary Table 2. Study population. Supplementary Table 3: Coverage details for CTSB. Supplemental Table 4: Primer and gRNA sequences used in the generation of CTSB-KO iPSC lines. Supplementary Table 5: Cell Culture Reagents and Media Compositions. Supplemental Table 6: CRISPRa and CRISPRi gRNA sequences. Supplemental Table 7: Antibodies. Supplemental Table 8: qPCR Primer sequences. Supplementary Table 9: GCTA-COLOC results. Supplementary Table 10: FINEMAP results of the nominated variants in *CTSB. *Supplementary Table 11: CTSB locus SMR and HEIDI analyses. Supplemental Table 12: Burden analysis for CTSB rare variants. Supplemental Table 13: gnomAD CTSB LOF. Supplemental Table 14: gnomAD SV CTSB.

Supplementary Material 2. Supplemental Figure 1: A) Immunofluorescent staining of parental (AIW002-02) and SNCA-KO iPSCs for pluripotency markers. B) Karyotype analysis of SNCA-KO iPSCs. C) Genome stability analysis of SNCA-KO iPSCs. D) Protein levels of alpha-synuclein in control versus SNCA-KO iPSCs after differentiation into dopaminergic neurons. E) Pluripotency marker expression in CTSB-KO iPSCs. F) Karyotype analysis of CTSB-KO iPSCs. G) Genome stability analysis of CTSB-KO iPSCs. H) Protein levels of cathepsin B in control versus CTSB-KO iPSCs after differentiation into dopaminergic neurons. Supplemental Figure 2:A) Differentiation of AIW002-2 iPSCs into dopaminergic neurons labelled for Map2 and tyrosine hydroxylase (TH). B) Percentage of TH-positive cells at 2 , 4, 6 and 8 weeks of differentiation. C) Representative electron microscopy imaging of a-syn PFFs. D) Measurement of a-syn PFF fibril size. Supplementary Figure 3: Immunofluorescent characterization of 5-month old midbrain organoids generated from SNCA triplication or SNCA-KO iPSCs. A-C) Markers of specific cell types present in SNCA triplication midbrain organoids including dopaminergic neurons (panel A, TH and GIRK2), inhibitory neurons (panel B, Map2 and GAD67), and astrocytes (panel C, GFAP). Oligodendrocytes (OLIG2) were not detected. D, E) Representative immunofluorescent images depicting a-syn expression in either SNCA triplication or SNCA-KO organoids demonstrating specificity of a-syn detection. Nuclei are indicated in blue (Hoechst), total neuronal content indicated in yellow (Map2), dopaminergic neurons are stained in red (TH – tyrosine hydroxylase), and a-syn is shown in green.
