# Correction: The Parkinson’s disease risk gene cathepsin B promotes fibrillar alpha-synuclein clearance, lysosomal function and glucocerebrosidase activity in dopaminergic neurons

**DOI:** 10.1186/s13024-024-00791-z

**Published:** 2024-12-18

**Authors:** Jace Jones-Tabah, Kathy He, Nathan Karpilovsky, Konstantin Senkevich, Ghislaine Deyab, Isabella Pietrantonio, Thomas Goiran, Yuting Cousineau, Daria Nikanorova, Taylor Goldsmith, Esther del Cid Pellitero, Carol X.-Q. Chen, Wen Luo, Zhipeng You, Narges Abdian, Jamil Ahmad, Jennifer A. Ruskey, Farnaz Asayesh, Dan Spiegelman, Stanley Fahn, Cheryl Waters, Oury Monchi, Yves Dauvilliers, Nicolas Dupré, Irina Miliukhina, Alla Timofeeva, Anton Emelyanov, Sofya Pchelina, Lior Greenbaum, Sharon Hassin-Baer, Roy N. Alcalay, Austen Milnerwood, Thomas M. Durcan, Ziv Gan-Or, Edward A. Fon

**Affiliations:** 1 Neurodegenerative Diseases Group, Department of Neurology and Neurosurgery, McGill Parkinson Program, Montreal Neurological Institute-Hospital, McGill University, Montreal, Québec Canada; 2 Department of Neurology and Neurosurgery, McGill University, Montréal, Canada; 3 Early Drug Discovery Unit (EDDU), Montreal Neurological Institute-Hospital, McGill University, Montreal, Canada; 4 Research Department, Bioinformatics Institute, Saint-Petersburg, Russia; 5 Department of Human Genetics, McGill University, Montréal, Canada; 6 Department of Neurology, College of Physicians and Surgeons, Columbia University Medical Center, New York, NY USA; 7 Département de Radiologie, Radio-Oncologie Et Médecine Nucléaire, Université de Montréal, Montréal, QC Canada; 8 Centre de Recherche de L’Institut Universitaire de Gériatrie de Montréal, Montréal, QC Canada; 9 Sleep Unit, Department of Neurology, National Reference Center for Narcolepsy, Gui-de-Chauliac Hospital, CHU Montpellier, University of Montpellier, Montpellier, France; 10 Neuroscience Axis, CHU de Québec – Université Laval, Quebec City, G1V 4G2 Canada; 11 Department of Medicine, Faculty of Medicine, Université Laval, Québec, QC G1V 0A6 Canada; 12 Institute of the Human Brain of RAS, St. Petersburg, Russia; 13 First Pavlov State Medical, University of St. Petersburg, Saint-Petersburg, Russia; 14 Sackler Faculty of Medicine, Tel Aviv University, Tel Aviv, Israel; 15 The Joseph Sagol Neuroscience Center, Sheba Medical Center, Tel Hashomer, Ramat Gan, Israel; 16 Department of Neurology, The Movement Disorders Institute, Sheba Medical Center, Tel Hashomer, Ramat Gan, Israel; 17 Neurological Institute, Tel Aviv Sourasky Medical Center, Tel Aviv, Israel

**Correction** **:** **Mol Neurodegeneration 19, 88 (2024)**

**https://doi.org/10.1186/s13024-024-00779-9**

The authors wish to note the Grant ID, MFFF – 007905 relating to the mentioned grants to ZGO and EAF from the Michael J. Fox Foundation which was mistakenly omitted from the original article [1].

The authors also wish to note that Lior Greenbaum is only affiliated to affiliation #14 (Sackler Faculty of Medicine, Tel Aviv University, Tel Aviv, Israel).
