# Supplementary material for: The Parkinson’s disease risk gene cathepsin B promotes fibrillar alpha-synuclein clearance, lysosomal function and glucocerebrosidase activity in dopaminergic neurons
Source: Mol Neurodegener. 2024 Nov 25;19:88. doi: 10.1186/s13024-024-00779-9 (PMC11587650; doi:10.1186/s13024-024-00779-9)

Supplemental Figure 1

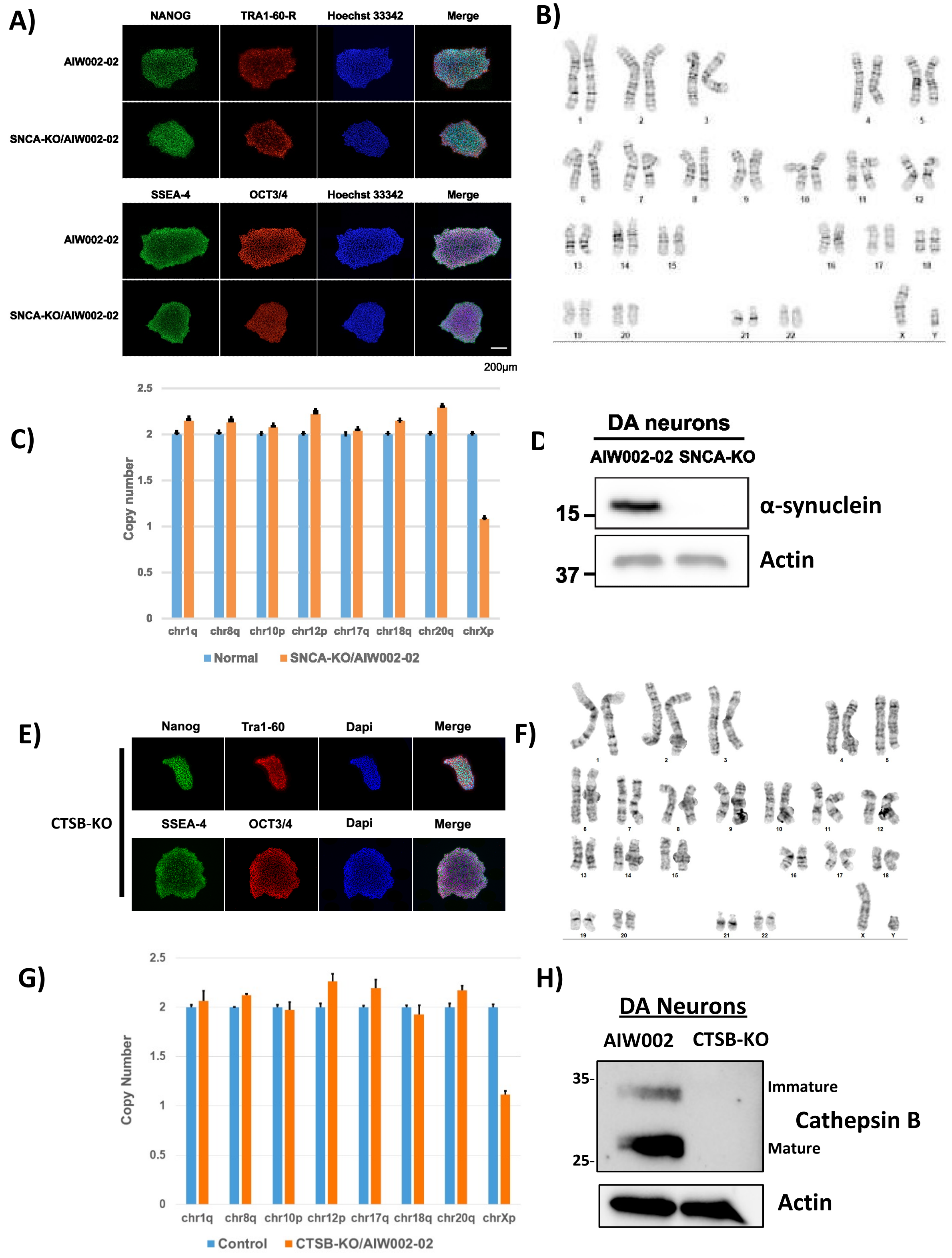

Supplementary Figure 2

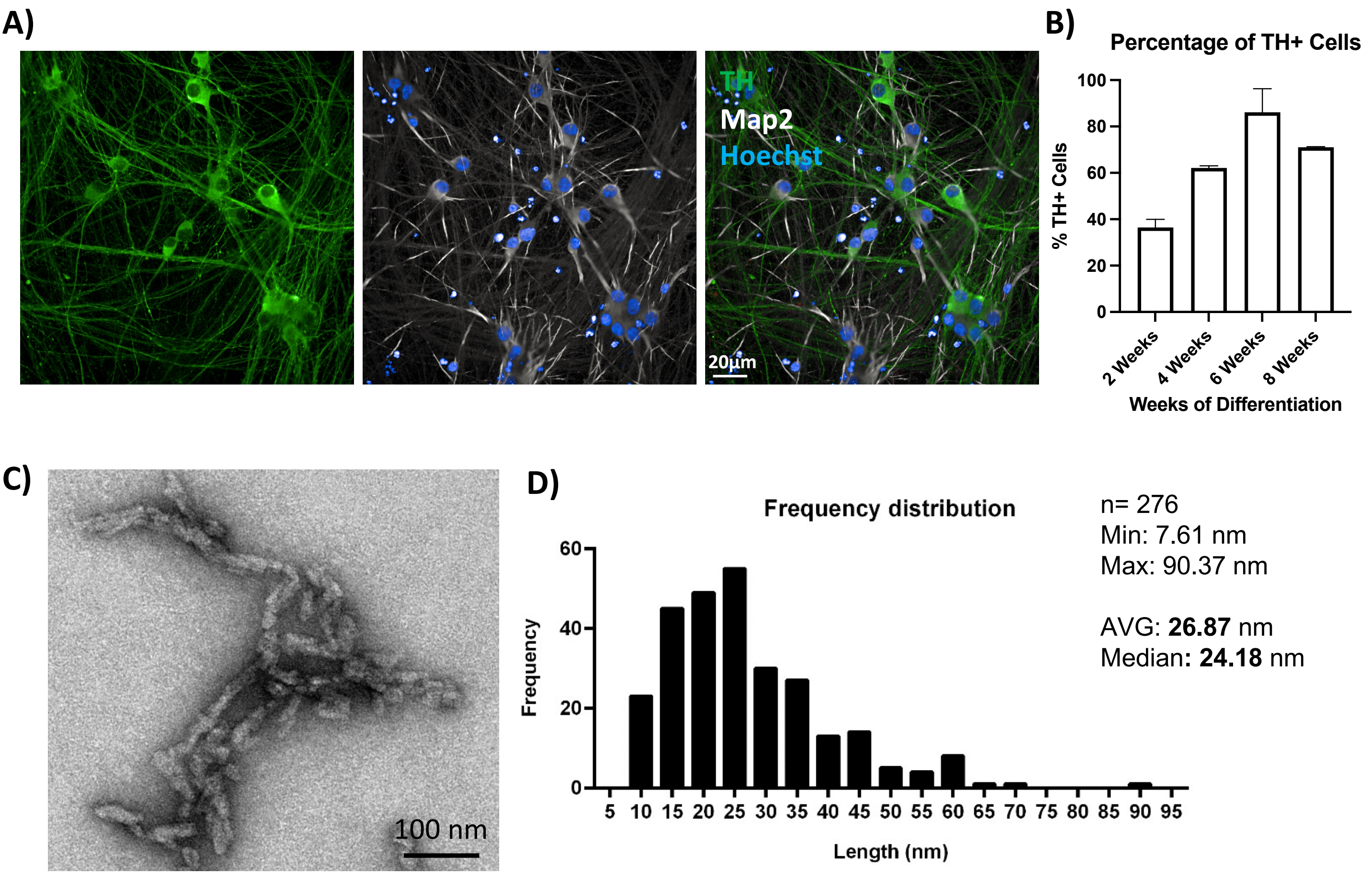

Supplementary Figure 3

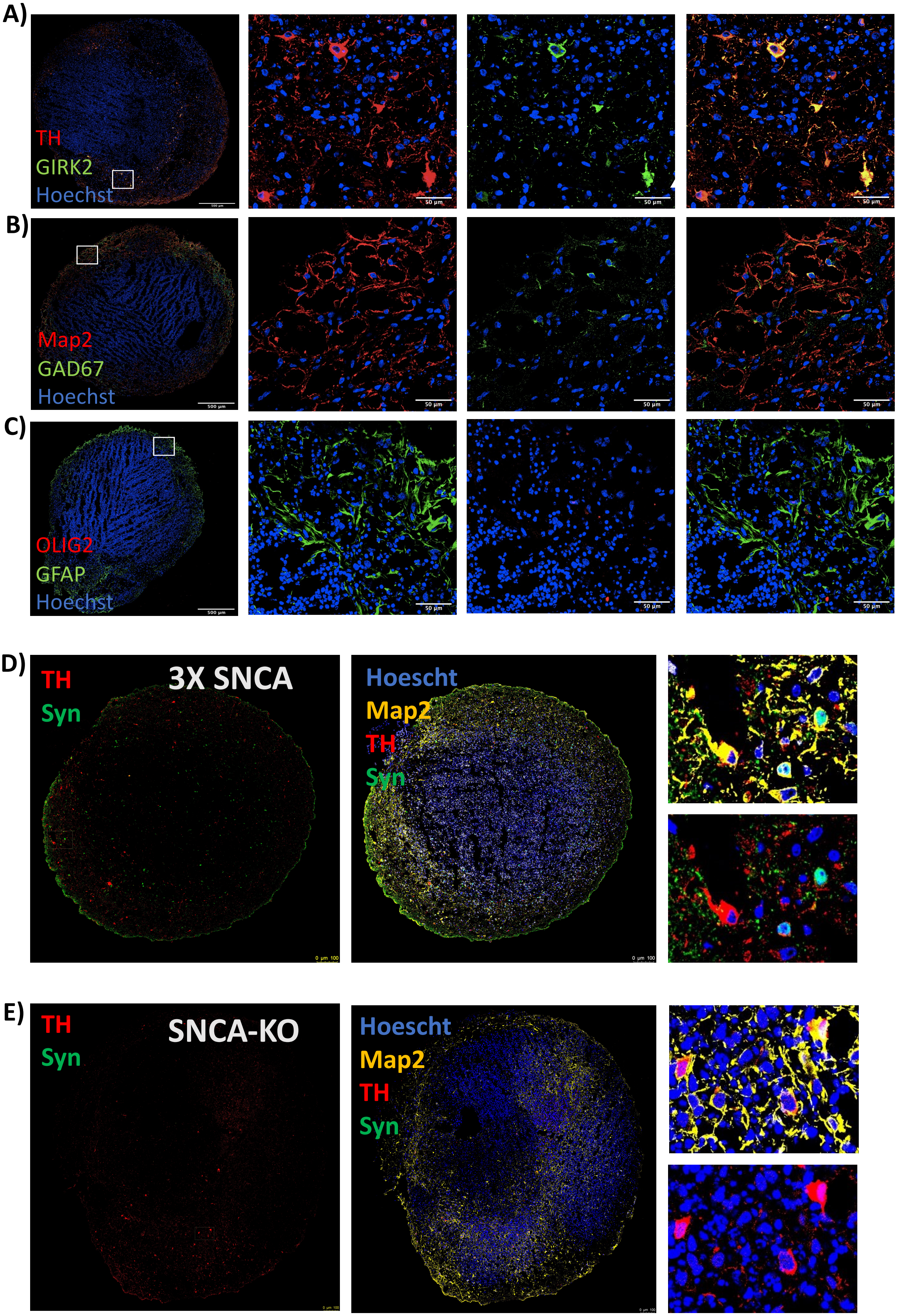

Figure 1

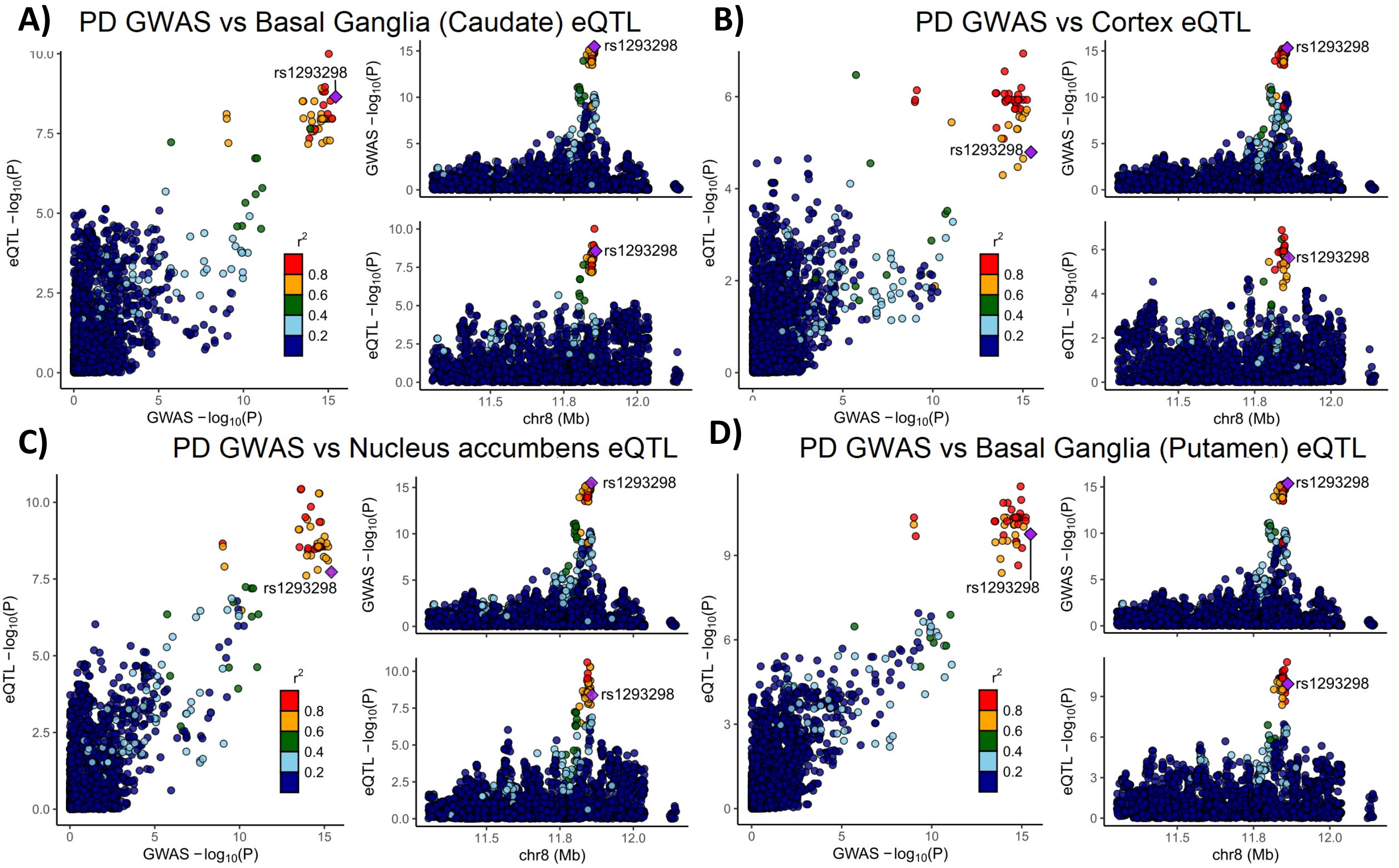

A)
PD GWAS vs Basal Ganglia (Caudate) eQTL
B)
PD GWAS vs Cortex eQTL
C)
PD GWAS vs Nucleus accumbens eQTL
D)
PD GWAS vs Basal Ganglia (Putamen) eQTL
rs1293298
eQTL −log10(P)
GWAS −log10(P)
chr8 (Mb)
r2
0.8
0.6
0.4
0.2

Figure 2

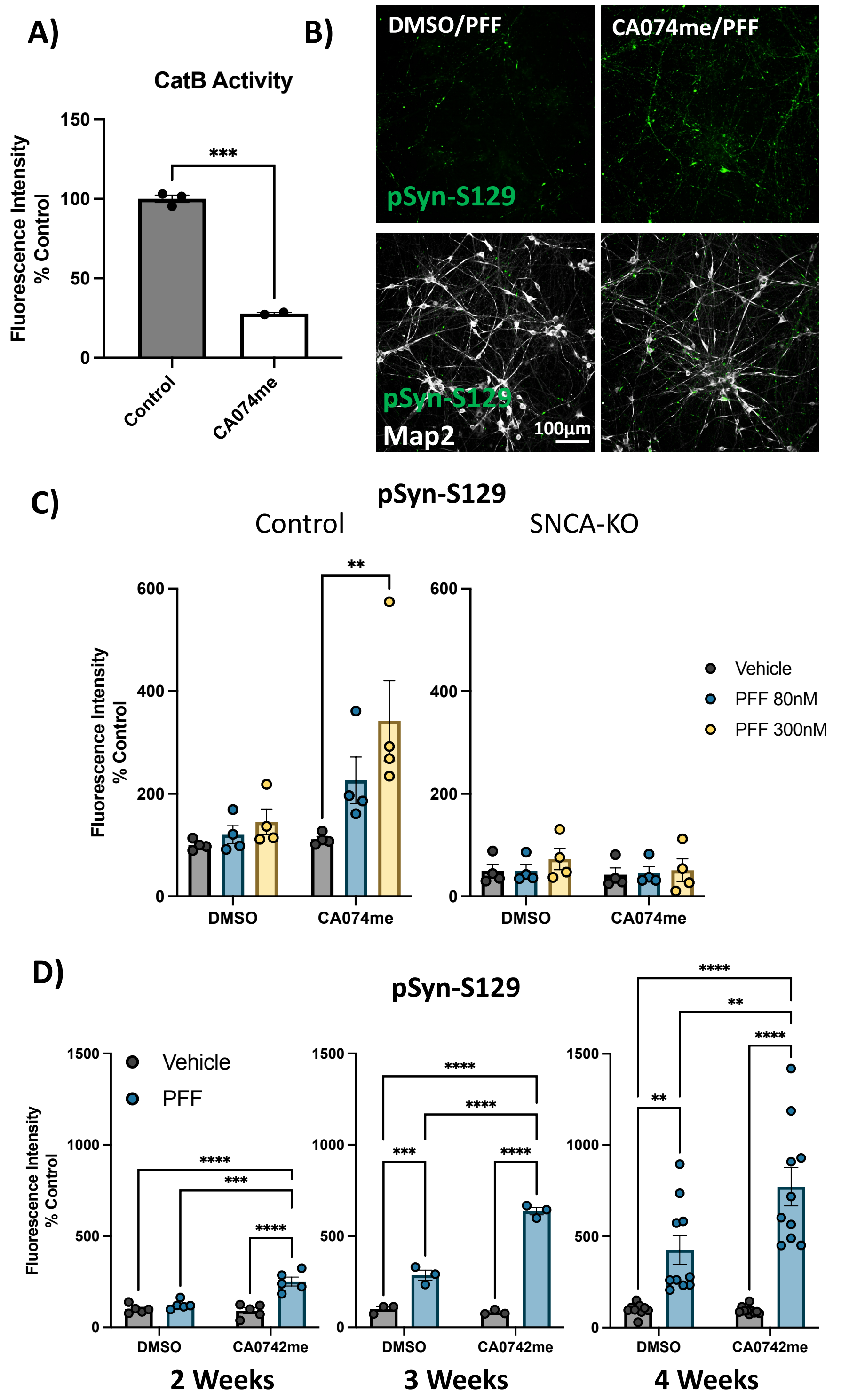
A)
CatB Activity
Fluorescence Intensity % Control
150
100
50
0
***
Control
CA074me
B)
DMSO/PFF
CA074me/PFF
pSyn-S129
pSyn-S129
Map2
100µm
C)
pSyn-S129
Control
SNCA-KO
Fluorescence Intensity % Control
600
400
200
0
**
DMSO
CA074me
Vehicle
PFF 80nM
PFF 300nM
D)
pSyn-S129
Vehicle
PFF
Fluorescence Intensity % Control
1500
1000
500
0
****
***
**
DMSO
CA0742me
2 Weeks
3 Weeks
4 Weeks

Supplemental Figure 4

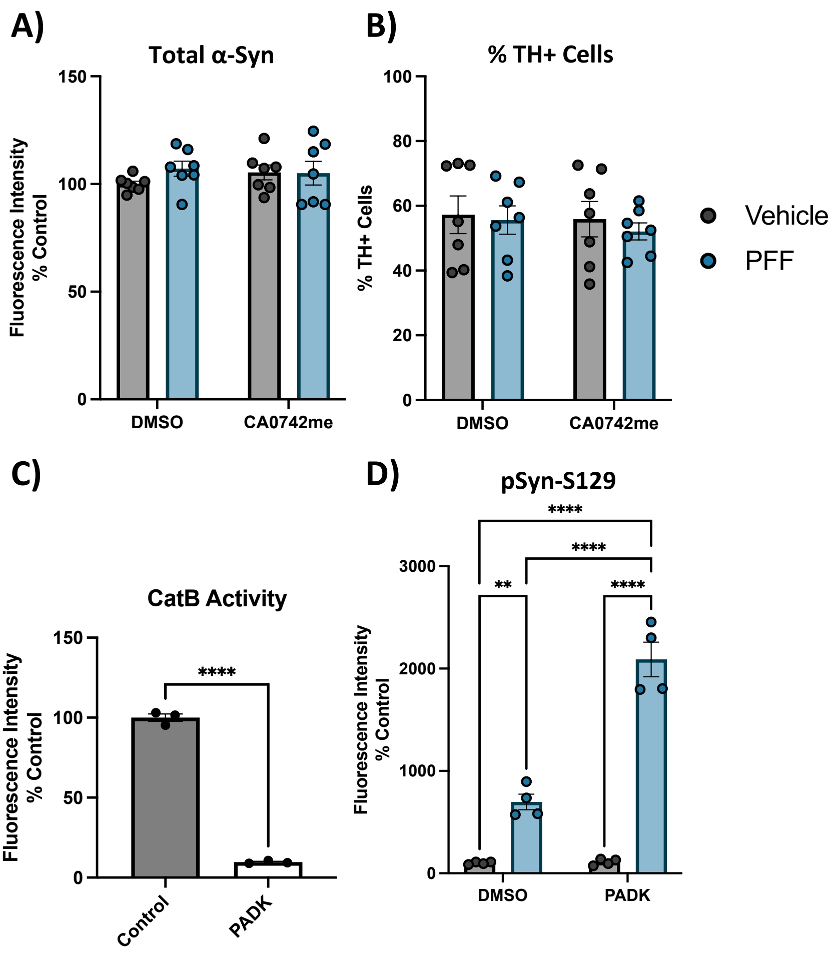

Figure 3

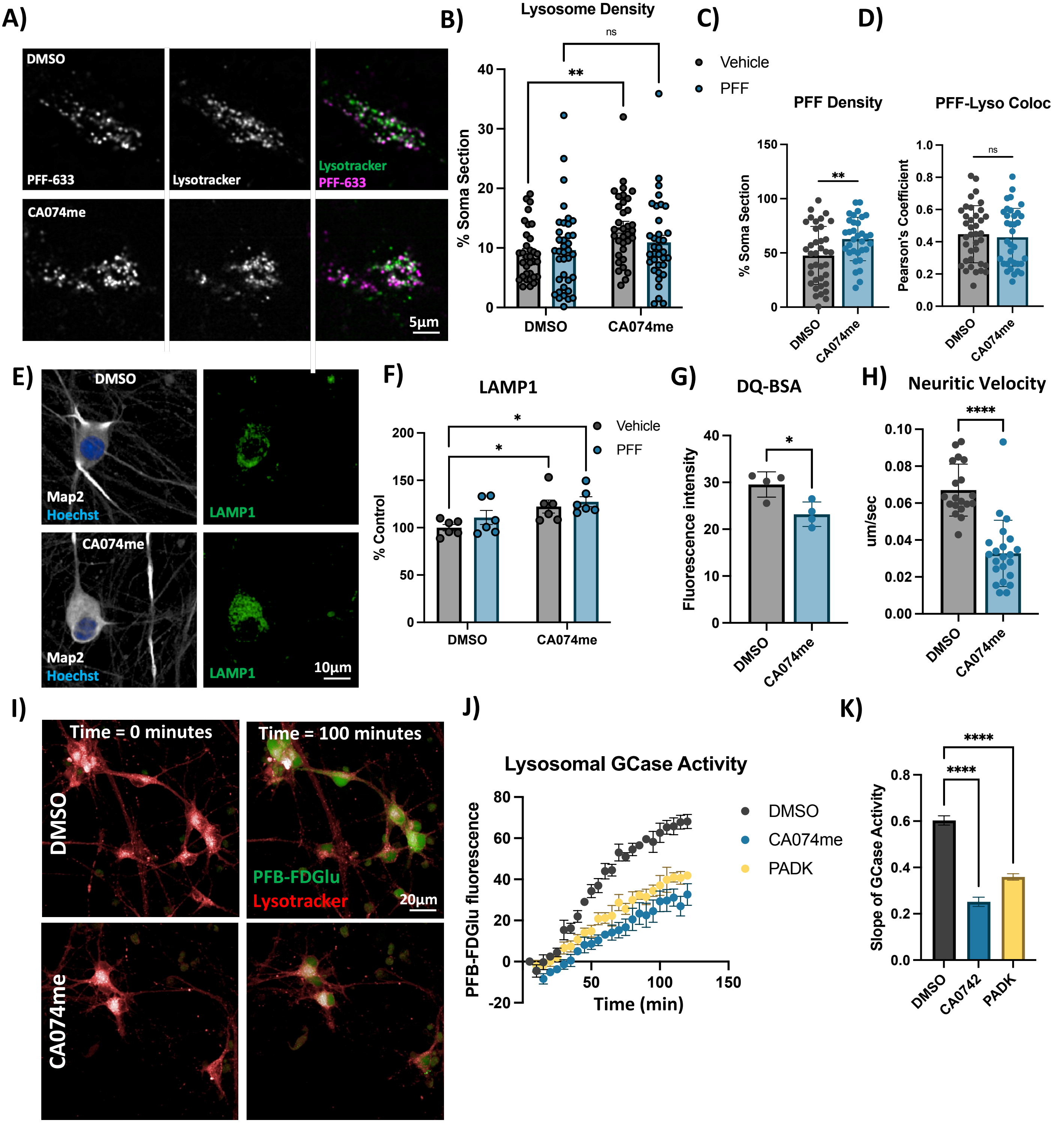
A)
DMSO
PFF-633
Lysotracker
Lysotracker
PFF-633
CA074me
5µm
B)
Lysosome Density
ns
**
% Soma Section
DMSO
CA074me
C)
Vehicle
PFF
PFF Density
**
% Soma Section
DMSO
CA074me
D)
PFF-Lyso Coloc
ns
Pearson's Coefficient
DMSO
CA074me
E)
DMSO
Map2
Hoechst
LAMP1
CA074me
Map2
Hoechst
LAMP1
10µm
F)
LAMP1
Vehicle
PFF
% Control
DMSO
CA074me
G)
DQ-BSA
Fluorescence intensity
DMSO
CA074me
H)
Neuritic Velocity
****
um/sec
DMSO
CA074me
I)
Time = 0 minutes
Time = 100 minutes
DMSO
CA074me
PFB-FDGlu
Lysotracker
20µm
J)
Lysosomal GCase Activity
PFB-FDGlu fluorescence
Time (min)
DMSO
CA074me
PADK
K)
****
****
Slope of GCase Activity
DMSO
CA0742
PADK

Supplemental Figure 5

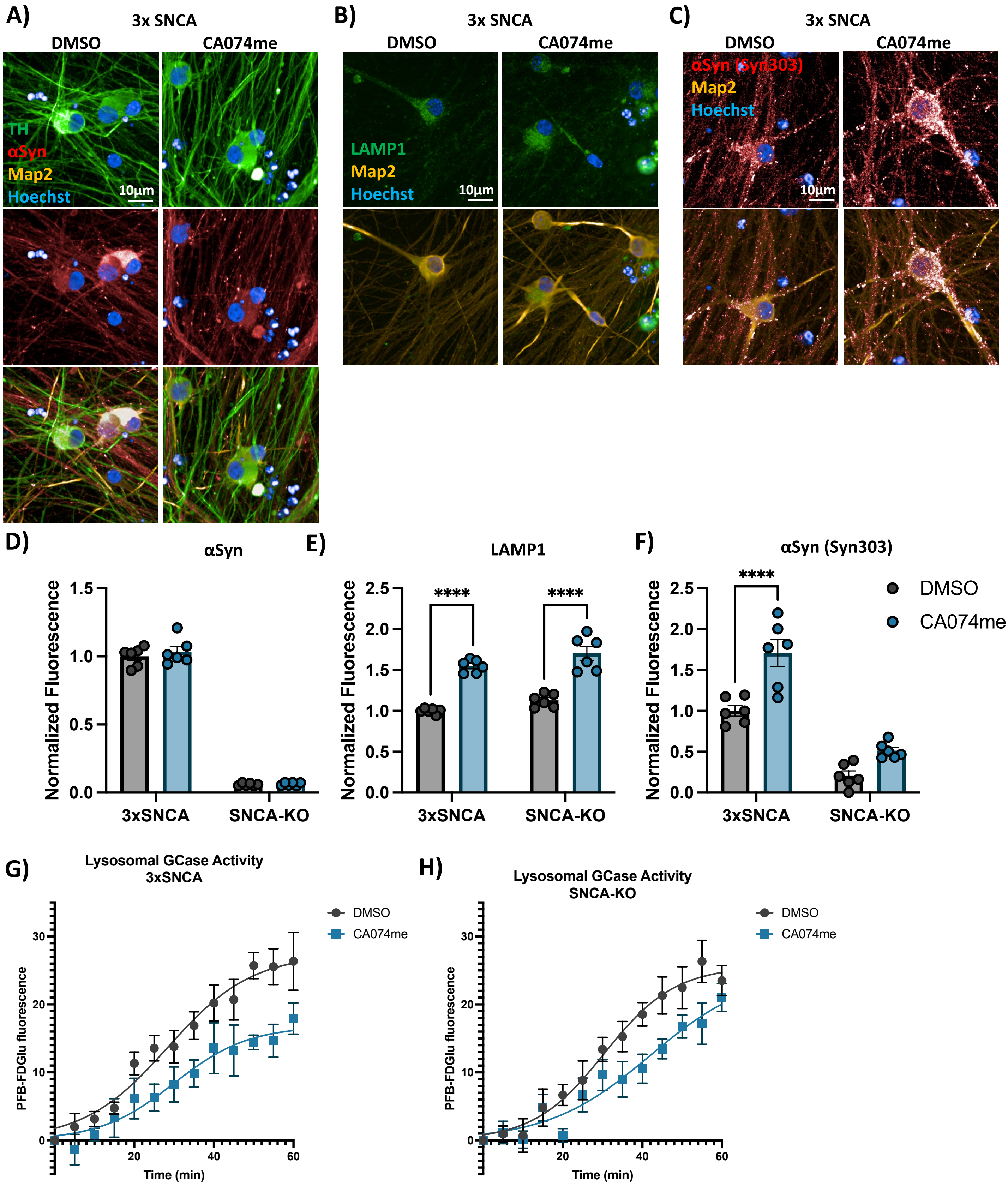

Figure 4

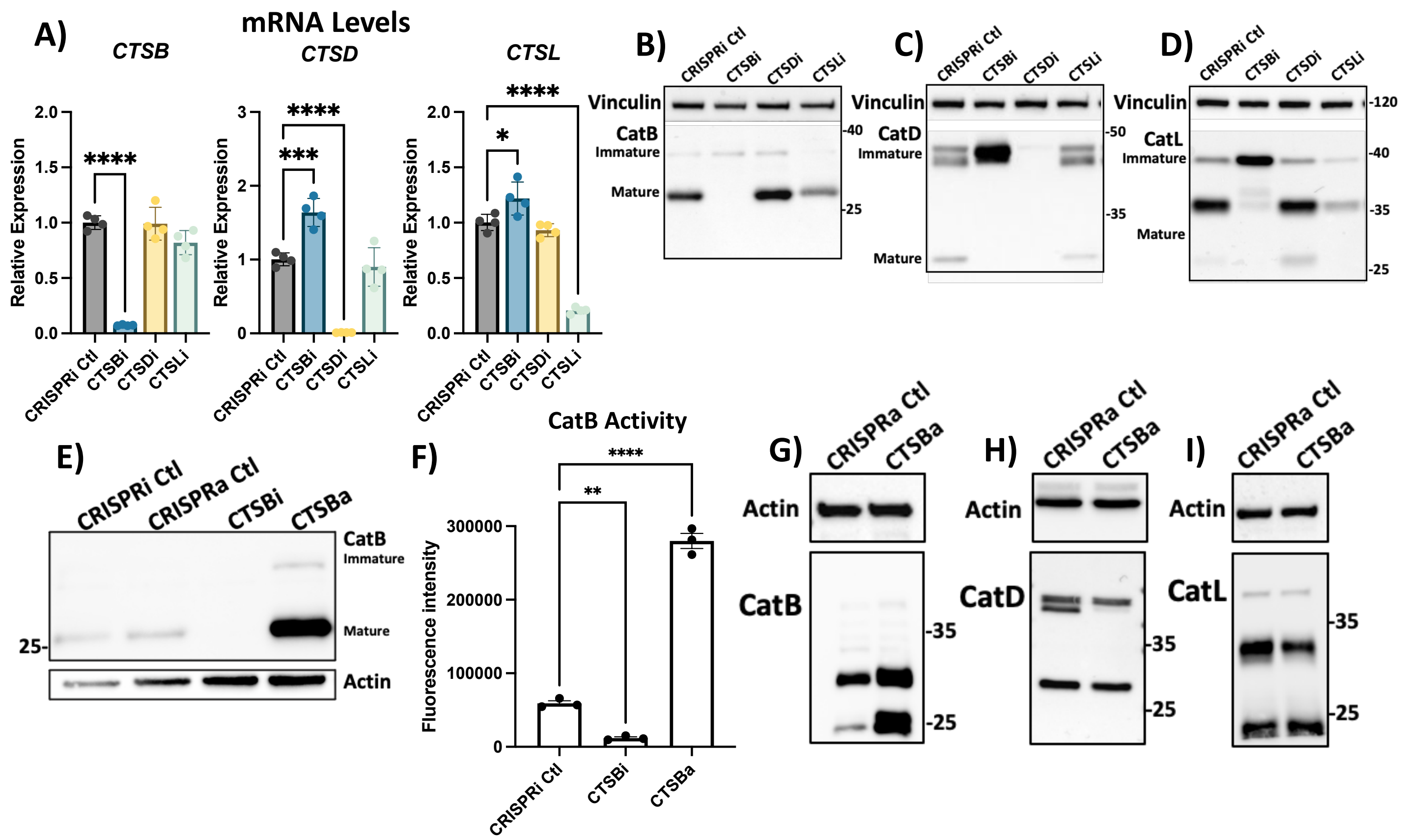
A)
mRNA Levels
CTSB
CTSD
CTSL
Relative Expression
CRISPRi Ctl
CTSBi
CTSDi
CTSLi
****
***
*
B)
C)
D)
Vinculin
CatB
CatD
CatL
Immature
Mature
-120
-50
-40
-35
-25
E)
CRISPRi Ctl
CRISPRa Ctl
CTSBi
CTSBa
Actin
25-
F)
CatB Activity
Fluorescence intensity
300000
200000
100000
0
**
G)
H)
I)
CRISPRa Ctl
CTSBa

Figure 5

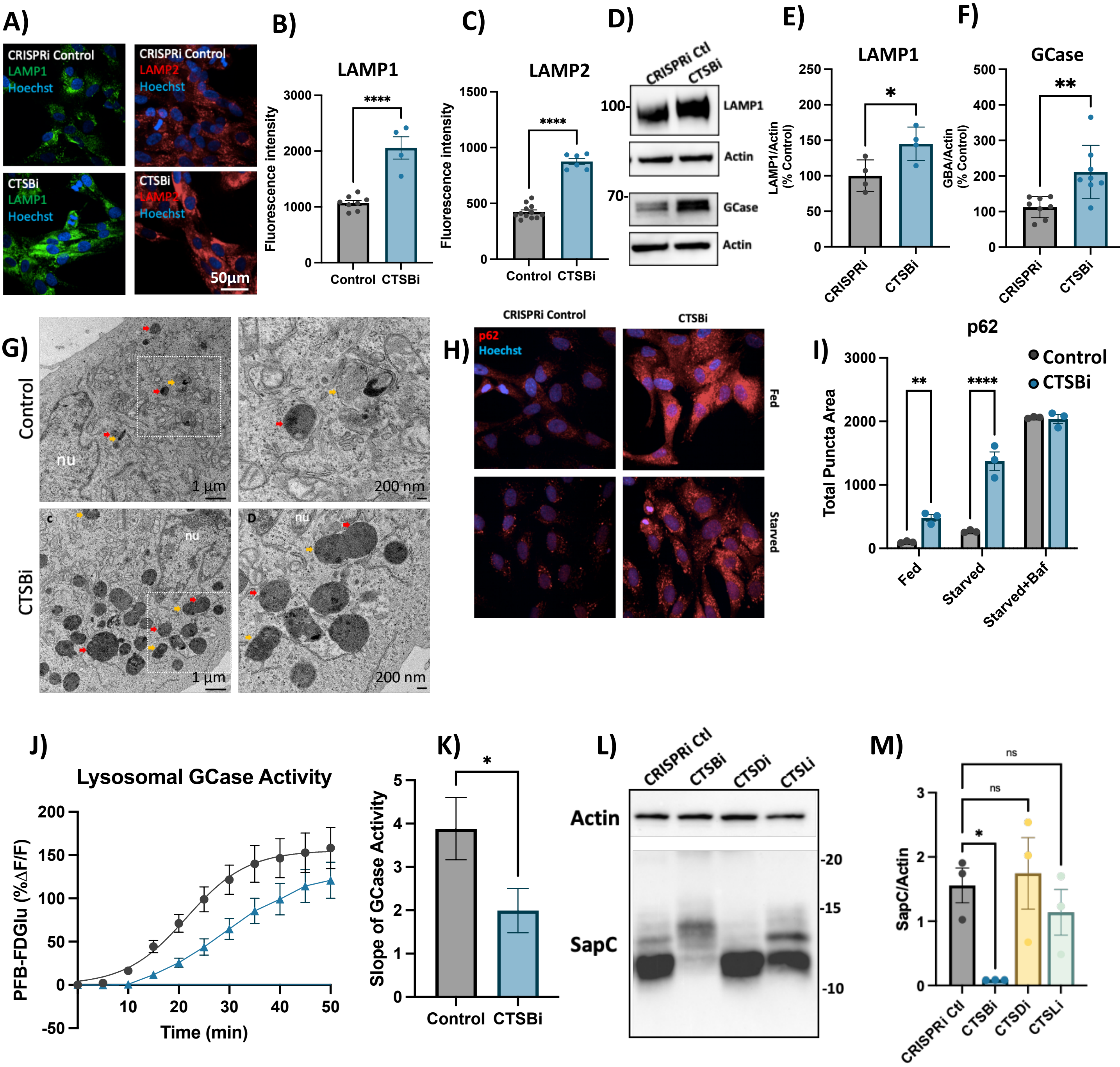

A)
CRISPRi Control
LAMP1
Hoechst
CRISPRi Control
LAMP2
Hoechst
CTSBi
LAMP1
Hoechst
CTSBi
LAMP2
Hoechst
50µm
B)
LAMP1
Fluorescence intensity
Control CTSBi
C)
LAMP2
Fluorescence intensity
Control CTSBi
D)
CRISPRi Ctl
CTSBi
LAMP1
Actin
GCase
Actin
E)
LAMP1
LAMP1/Actin (% Control)
CRISPRi
CTSBi
F)
GCase
GBA/Actin (% Control)
CRISPRi
CTSBi
G)
Control
CTSBi
nu
1 µm
200 nm
H)
CRISPRi Control
CTSBi
p62
Hoechst
Fed
Starved
I)
p62
Total Puncta Area
Control
CTSBi
Fed
Starved
Starved+Baf
J)
Lysosomal GCase Activity
PFB-FDGlu (%ΔF/F)
Time (min)
K)
Slope of GCase Activity
Control CTSBi
L)
CRISPRi Ctl
CTSBi
CTSDi
CTSLi
Actin
SapC
M)
SapC/Actin
ns
CRISPRi Ctl
CTSBi
CTSDi
CTSLi

Supplemental Figure 6:

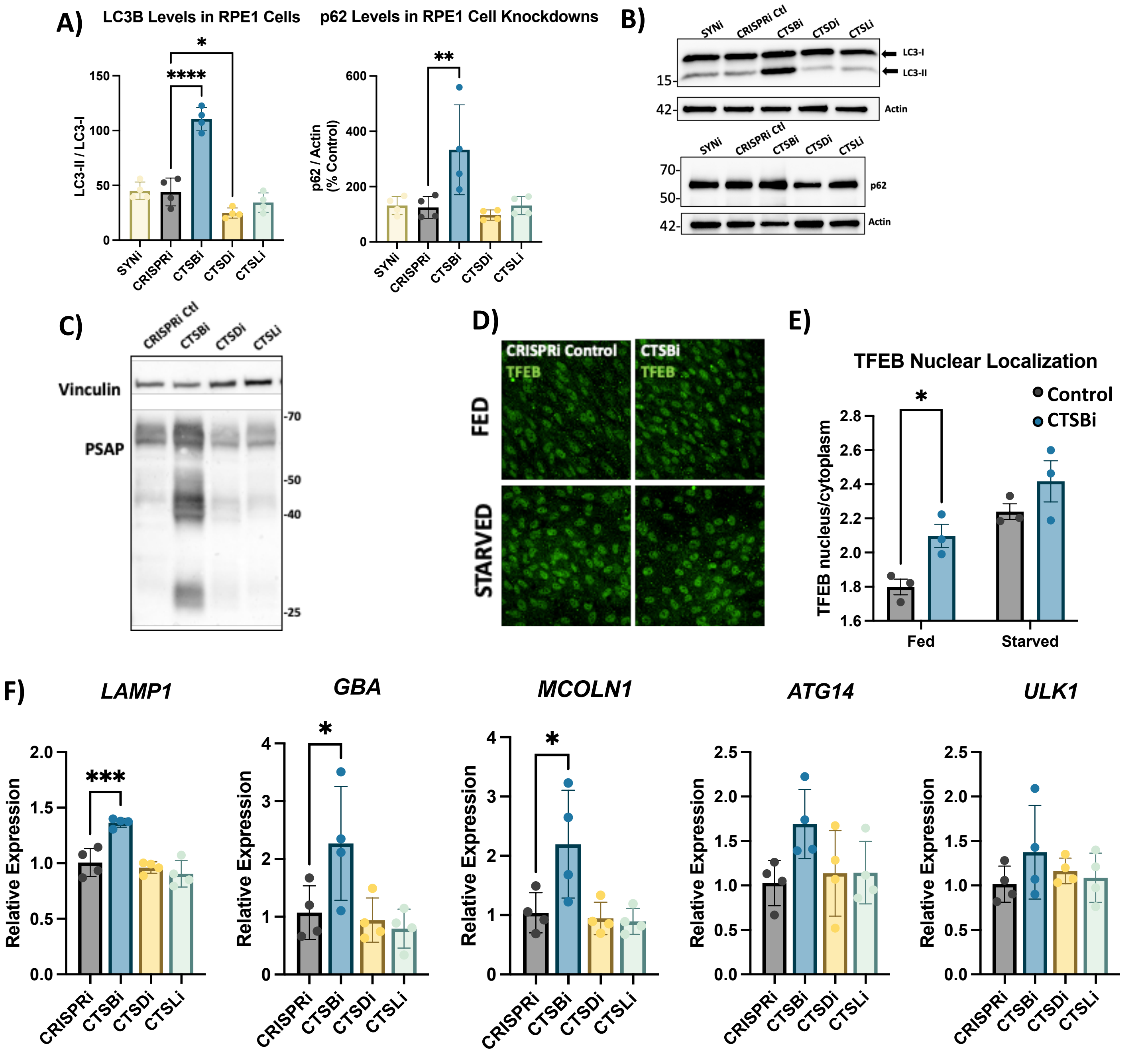

Figure 6

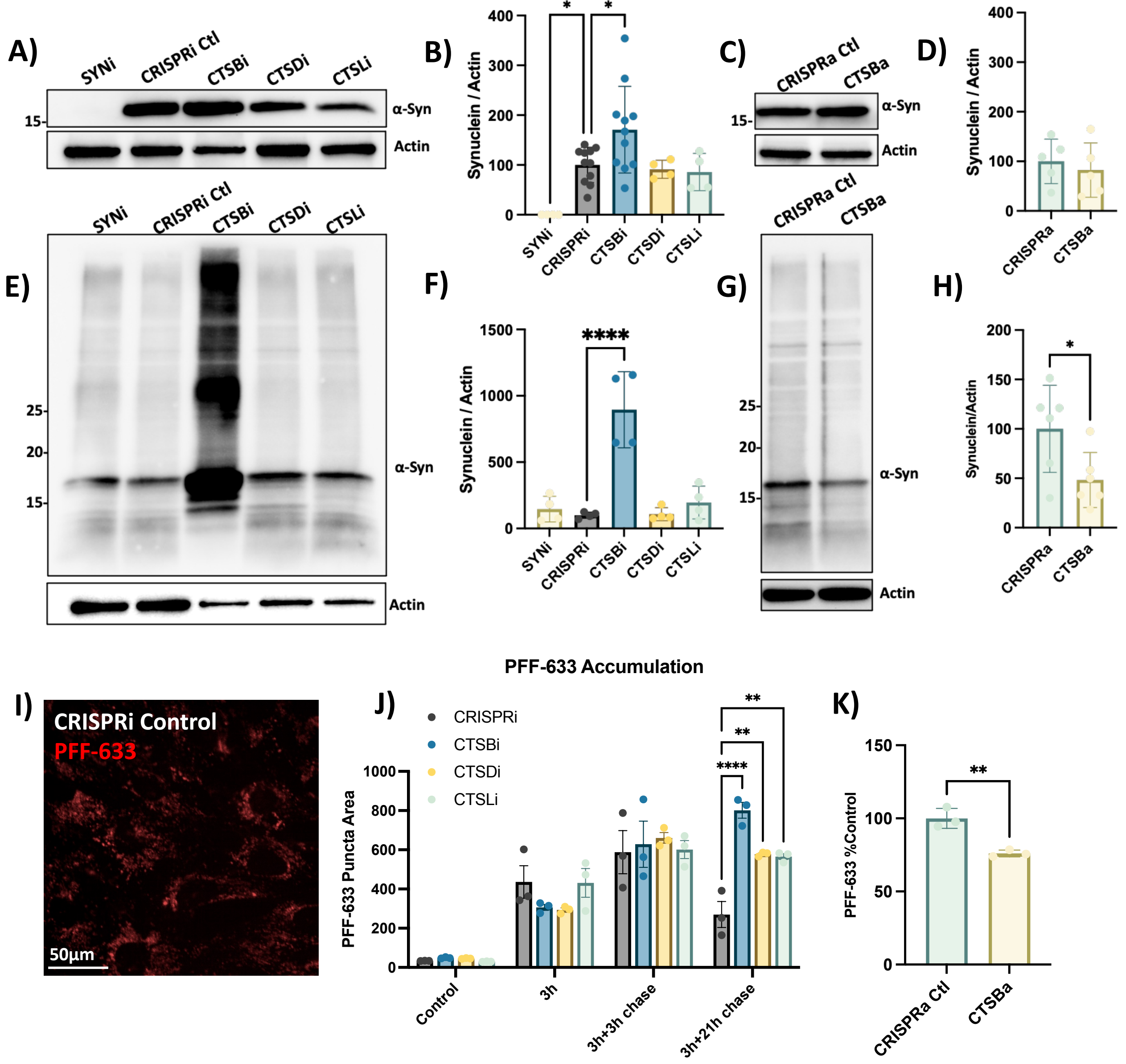
A)
SYNi
CRISPRi Ctl
CTSBi
CTSDi
CTSLi
15-
α-Syn
Actin
B)
Synuclein / Actin
400
300
200
100
0
*
*
SYNi
CRISPRi
CTSBi
CTSDi
CTSLi
C)
CRISPRa Ctl
CTSBa
15-
α-Syn
Actin
D)
Synuclein / Actin
400
300
200
100
0
CRISPRa
CTSBa
E)
SYNi
CRISPRi Ctl
CTSBi
CTSDi
CTSLi
25-
20-
15-
α-Syn
Actin
F)
Synuclein / Actin
1500
1000
500
0
****
SYNi
CRISPRi
CTSBi
CTSDi
CTSLi
G)
CRISPRa Ctl
CTSBa
25-
20-
15-
α-Syn
Actin
H)
Synuclein/Actin
200
150
100
50
0
*
CRISPRa
CTSBa
I)
CRISPRi Control
PFF-633
50µm
PFF-633 Accumulation
J)
CRISPRi
CTSBi
CTSDi
CTSLi
PFF-633 Puncta Area
1000
800
600
400
200
0
**
**
****
Control
3h
3h+3h chase
3h+21h chase
K)
PFF-633 %Control
150
100
50
0
**
CRISPRa Ctl
CTSBa

Supplemental Figure 7

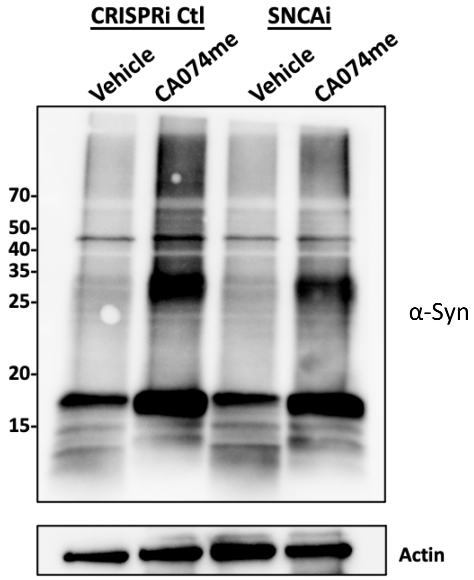

Figure 7

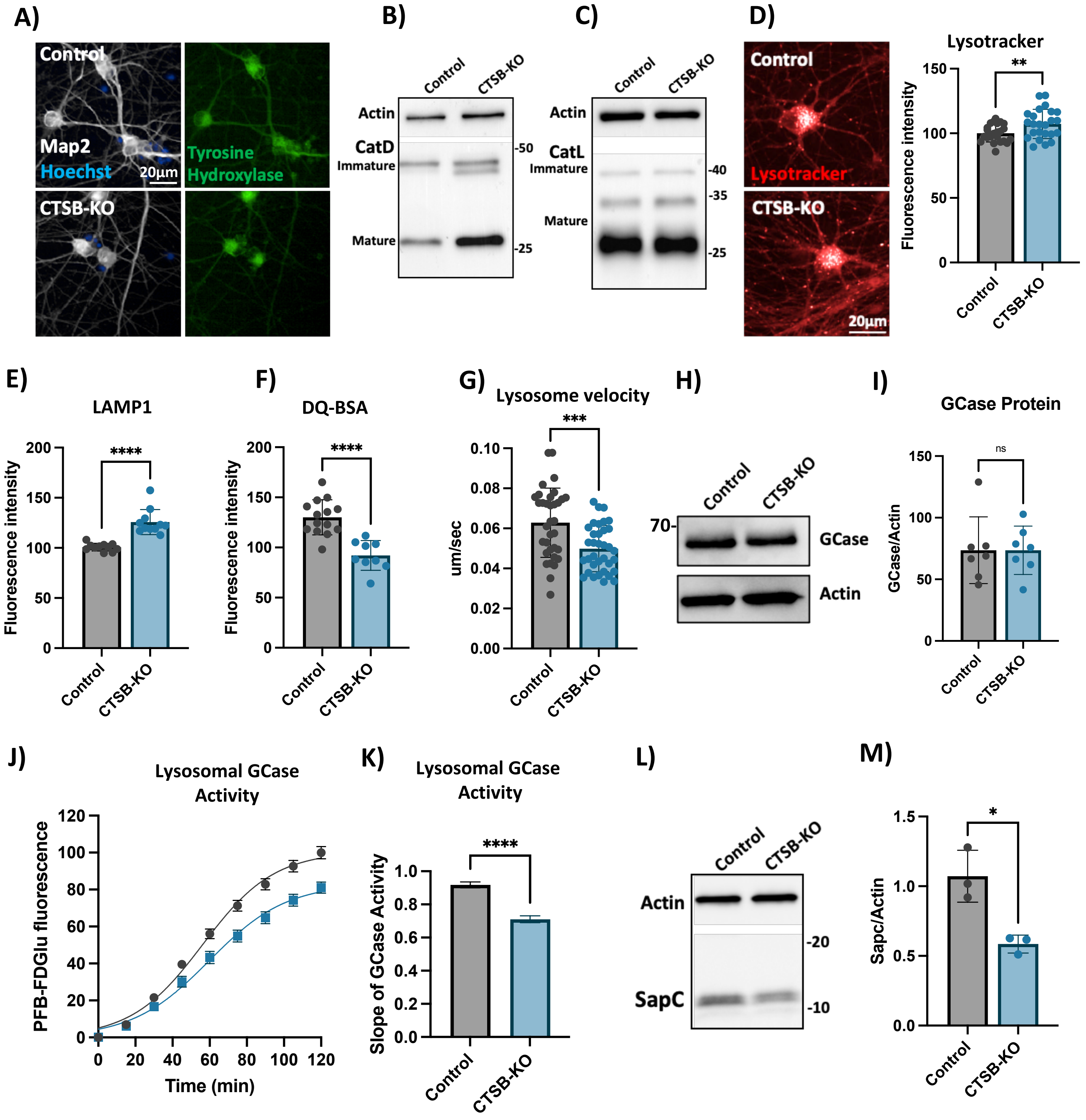

A)
Control
Map2
Hoechst
20µm
Tyrosine Hydroxylase
CTSB-KO
B)
Control
CTSB-KO
Actin
CatD
Immature
Mature
-50
-25
C)
Control
CTSB-KO
Actin
CatL
Immature
Mature
-40
-35
-25
D)
Control
Lysotracker
CTSB-KO
20µm
Lysotracker
Fluorescence intensity
**
150
100
50
0
E)
LAMP1
****
Fluorescence intensity
200
150
100
50
0
F)
DQ-BSA
****
Fluorescence intensity
G)
Lysosome velocity
***
um/sec
0.10
0.08
0.06
0.04
0.02
0.00
H)
70-
GCase
Actin
I)
GCase Protein
ns
GCase/Actin
J)
Lysosomal GCase Activity
PFB-FDGlu fluorescence
120
100
80
60
40
20
0
Time (min)
K)
Lysosomal GCase Activity
****
Slope of GCase Activity
1.0
0.8
0.6
0.4
0.2
0.0
L)
Actin
SapC
-20
-10
M)
*
Sapc/Actin
1.5
1.0
0.5
0.0

Supplemental Figure 8

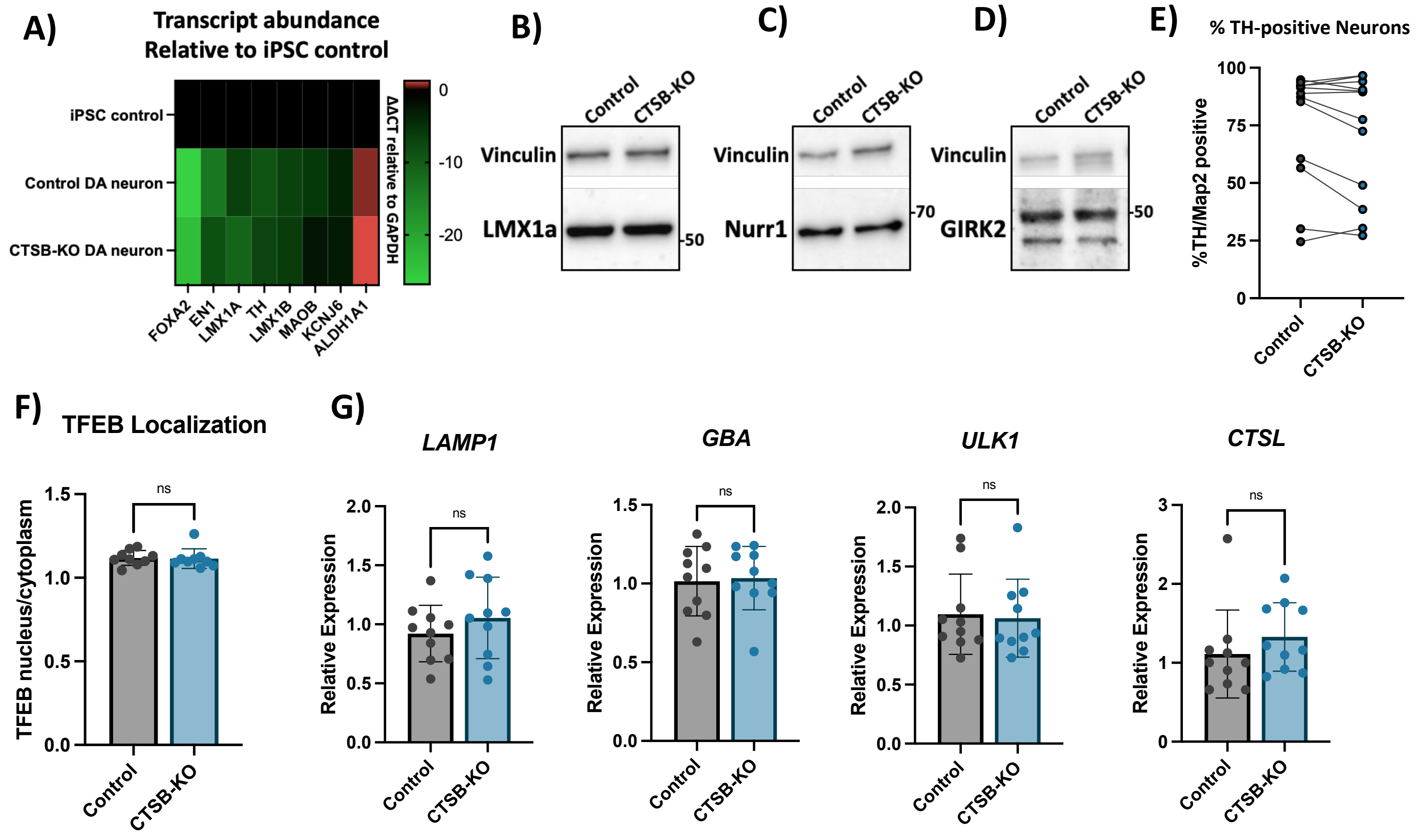

Figure 8

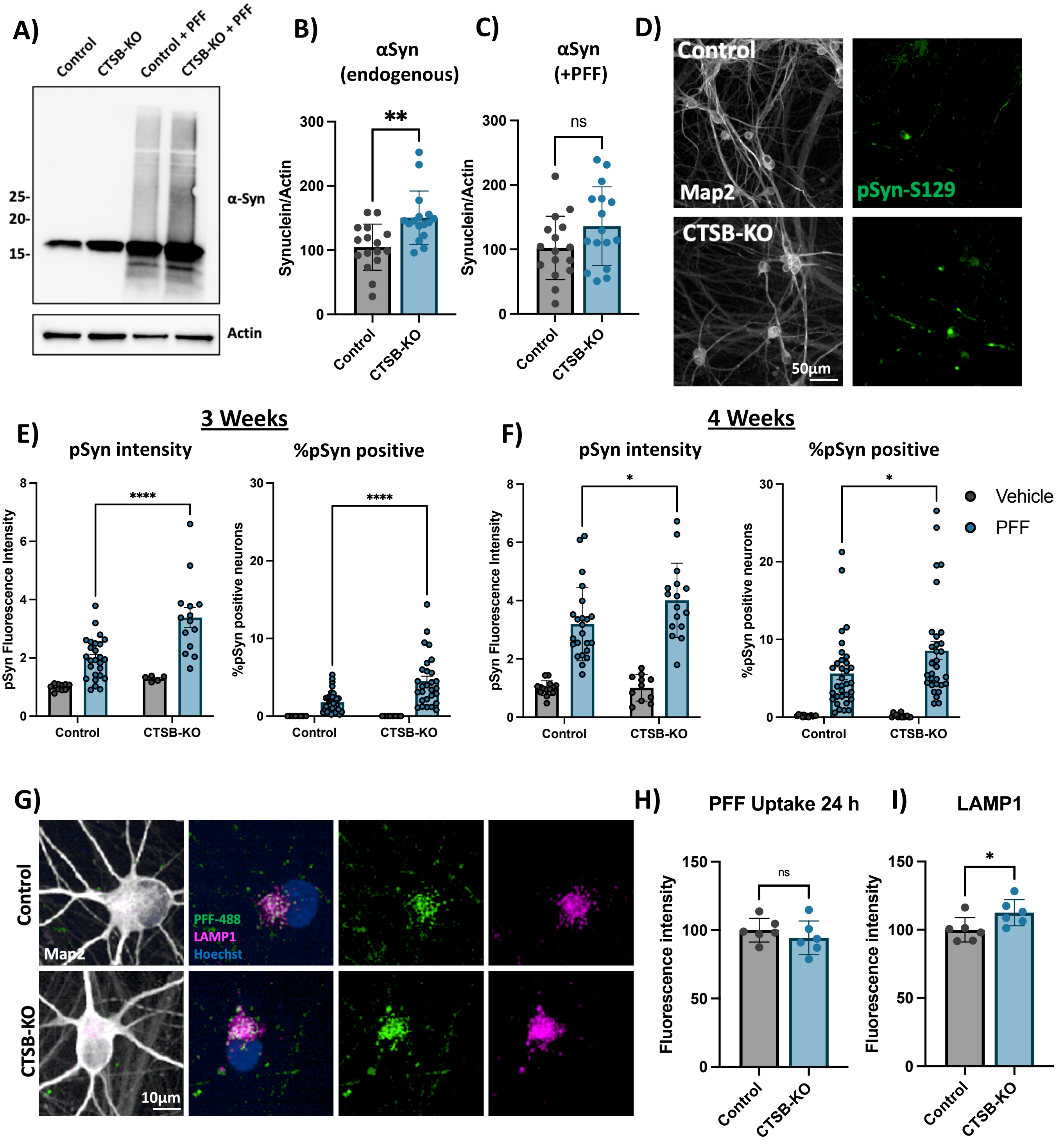

A)
Control
CTSB-KO
Control + PFF
CTSB-KO + PFF
25-
20-
15-
α-Syn
Actin
B)
αSyn (endogenous)
**
Synuclein/Actin
Control
CTSB-KO
C)
αSyn (+PFF)
ns
Synuclein/Actin
Control
CTSB-KO
D)
Control
Map2
pSyn-S129
CTSB-KO
50µm
E)
3 Weeks
pSyn intensity
****
pSyn Fluorescence Intensity
%pSyn positive
****
%pSyn positive neurons
Control
CTSB-KO
F)
4 Weeks
pSyn intensity
*
pSyn Fluorescence Intensity
%pSyn positive
*
%pSyn positive neurons
Control
CTSB-KO
Vehicle
PFF
G)
Control
CTSB-KO
Map2
PFF-488
LAMP1
Hoechst
10µm
H)
PFF Uptake 24 h
ns
Fluorescence intensity
Control
CTSB-KO
I)
LAMP1
*
Fluorescence intensity
Control
CTSB-KO

Supplemental Figure 9

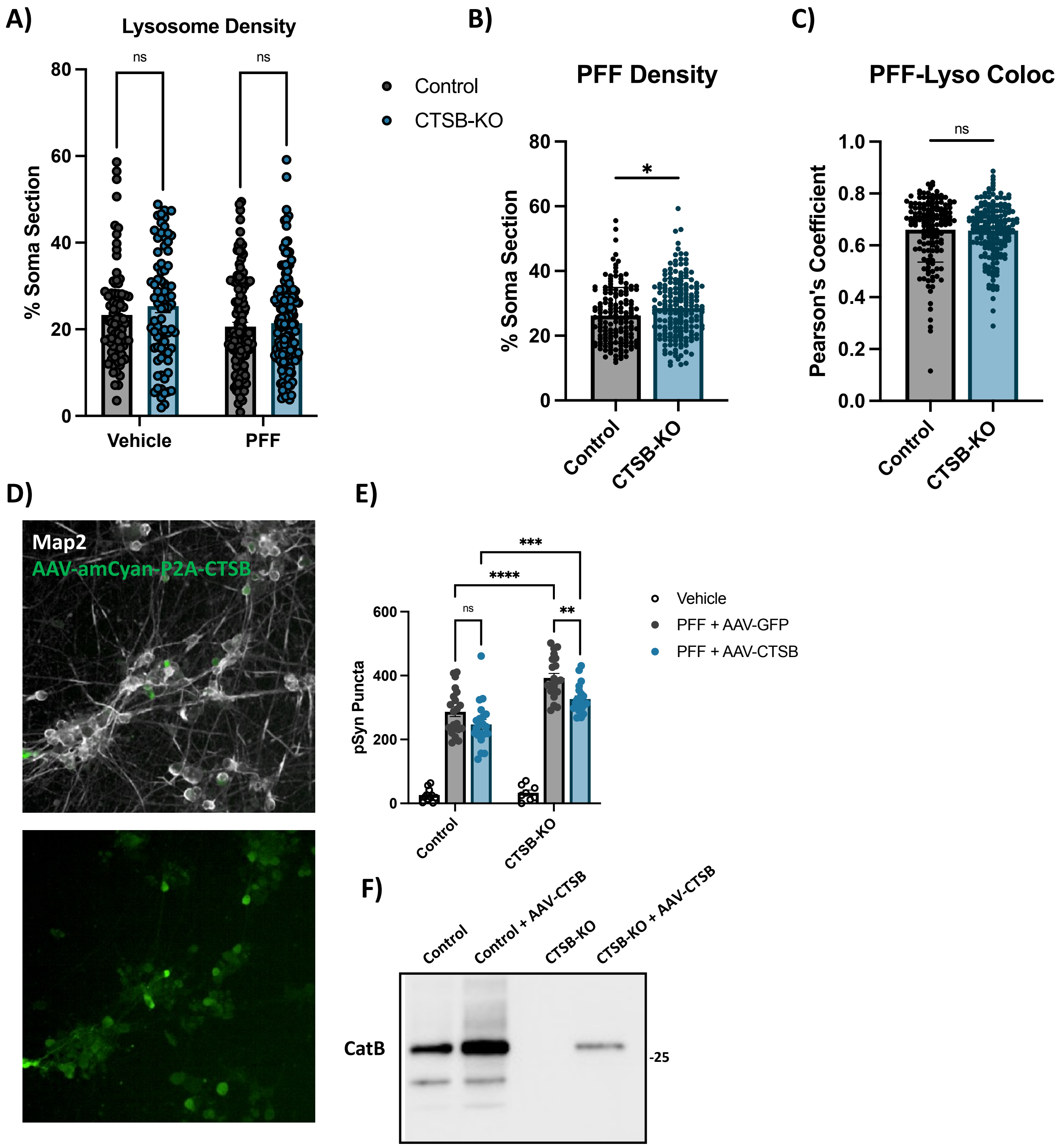

Figure 9

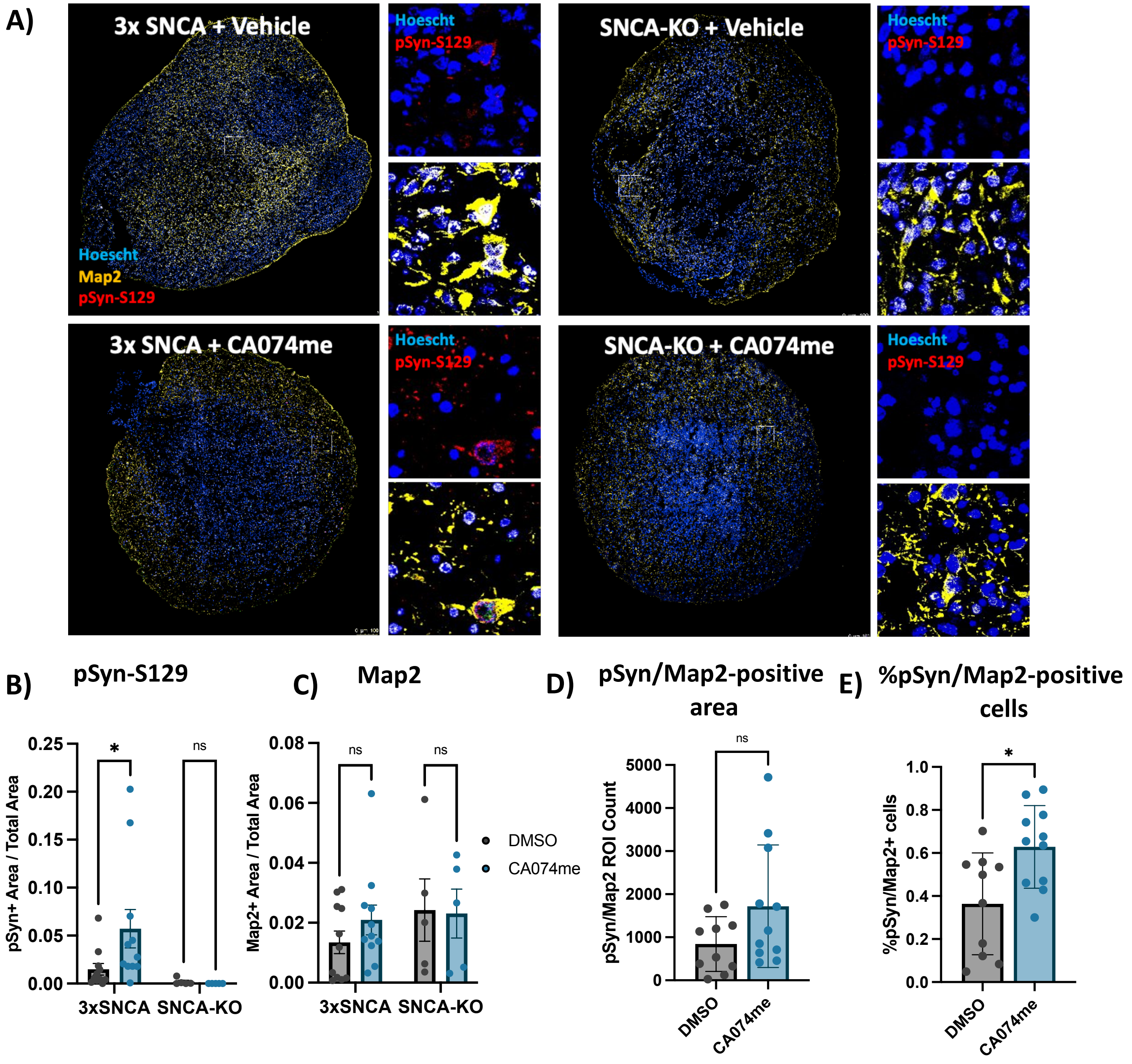
A)
3x SNCA + Vehicle
SNCA-KO + Vehicle
3x SNCA + CA074me
SNCA-KO + CA074me
Hoescht
Map2
pSyn-S129
B)
pSyn-S129
pSyn+ Area / Total Area
3xSNCA
SNCA-KO
*
ns
C)
Map2
Map2+ Area / Total Area
DMSO
CA074me
D)
pSyn/Map2-positive area
pSyn/Map2 ROI Count
E)
%pSyn/Map2-positive cells
%pSyn/Map2+ cells

Supplemental Figure 10

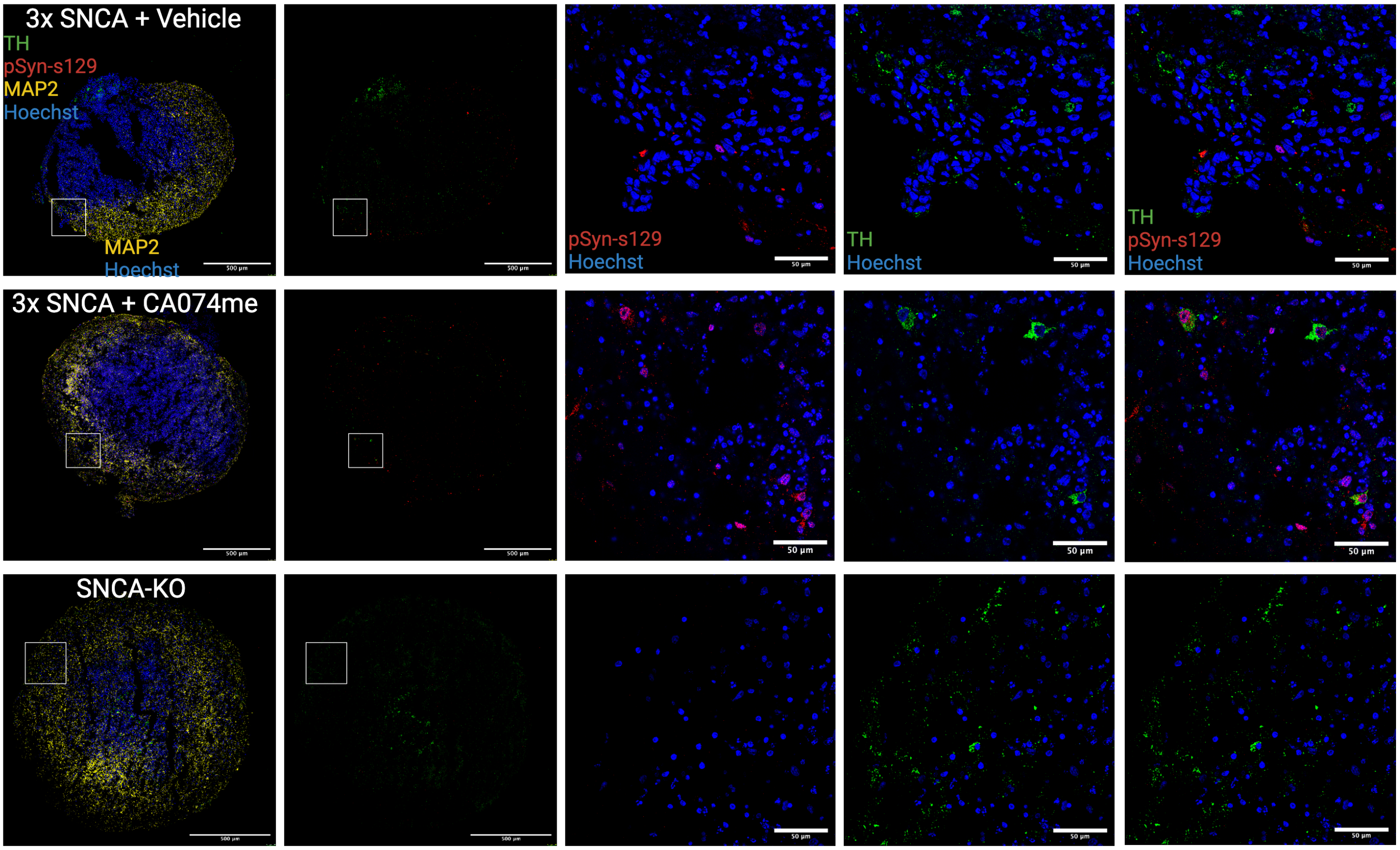

Supplement: Supplementary file 2 — Supplementary Material 2. Supplemental Figure 1: A) Immunofluorescent staining of parental (AIW002-02) and SNCA-KO iPSCs for pluripotency markers. B) Karyotype analysis of SNCA-KO iPSCs. C) Genome stability analysis of SNCA-KO iPSCs. D) Protein levels of alpha-synuclein in control versus SNCA-KO iPSCs after differentiation into dopaminergic neurons. E) Pluripotency marker expression in CTSB-KO iPSCs. F) Karyotype analysis of CTSB-KO iPSCs. G) Genome stability analysis of CTSB-KO iPSCs. H) Protein levels of cathepsin B in control versus CTSB-KO iPSCs after differentiation into dopaminergic neurons. Supplemental Figure 2:A) Differentiation of AIW002-2 iPSCs into dopaminergic neurons labelled for Map2 and tyrosine hydroxylase (TH). B) Percentage of TH-positive cells at 2 , 4, 6 and 8 weeks of differentiation. C) Representative electron microscopy imaging of a-syn PFFs. D) Measurement of a-syn PFF fibril size. Supplementary Figure 3: Immunofluorescent characterization of 5-month old midbrain organoids generated from SNCA triplication or SNCA-KO iPSCs. A-C) Markers of specific cell types present in SNCA triplication midbrain organoids including dopaminergic neurons (panel A, TH and GIRK2), inhibitory neurons (panel B, Map2 and GAD67), and astrocytes (panel C, GFAP). Oligodendrocytes (OLIG2) were not detected. D, E) Representative immunofluorescent images depicting a-syn expression in either SNCA triplication or SNCA-KO organoids demonstrating specificity of a-syn detection. Nuclei are indicated in blue (Hoechst), total neuronal content indicated in yellow (Map2), dopaminergic neurons are stained in red (TH – tyrosine hydroxylase), and a-syn is shown in green. [file 13024_2024_779_MOESM2_ESM.pdf]
